# Community health worker support to improve HIV treatment outcomes for older children and adolescents in Zimbabwe: a process evaluation of the ZENITH trial

**DOI:** 10.1186/s13012-018-0762-5

**Published:** 2018-05-23

**Authors:** Chido Dziva Chikwari, Victoria Simms, Joanna Busza, Ethel Dauya, Tsitsi Bandason, Prosper Chonzi, Shungu Munyati, Hilda Mujuru, Rashida A. Ferrand

**Affiliations:** 1 London School of Hygiene and Tropical Medicine, Keppel St, London, WC1E 7HT UK; 2 Biomedical Research and Training Institute, 10 Seagrave Road, Avondale, Harare, Zimbabwe; 3 City of Harare Health Services Department, Rowan Martin Building, Harare, Zimbabwe; 4 University of Zimbabwe College of Health Sciences, P O Box A178, Avondale, Harare, Zimbabwe

**Keywords:** HIV, Children, Community-based support, Adherence, ART

## Abstract

**Background:**

Community health worker (CHW)-delivered support visits to children living with HIV and their caregivers significantly reduced odds of virological failure among the children in the ZENITH trial conducted in Zimbabwe. We conducted a process evaluation to assess fidelity, acceptability, and feasibility of this intervention to identify lessons that could inform replication and scale-up of this approach.

**Methods:**

Field manuals kept by each CHW, records from monthly supervisory meetings, and participant data collected throughout the trial were used to assess the intervention’s implementation. Data extracted from field manuals included visit type, content, and duration. Minutes from monthly supervisory meetings were used to capture CHW attendance.

**Results:**

The trial enrolled 172 participants in the intervention arm of whom 5 subsequently refused all visits, 1 died before the intervention could be delivered, and 1 could not be located. Manuals for 8 participants were not returned, 3 were incorrectly entered, and 1 manual was lost. We had 154 manuals available for analysis.

A total of 1553 visits were successfully conducted (median 11 per participant, range 1–20). Additionally, CHWs made 85 visits where they were unable to make contact with the family. Thirteen (8.4%) participants received 5 or fewer visits, 10 moved out of the study area, and 3 died. CHWs discussed disclosure with the child/family for over 89% of participants and assisted clients with developing and reviewing their personal treatment plan with over 85% of participants. Of the 20 CHWs (3 male, 17 female) selected to implement the intervention, 19 were retained at the end of the trial.

**Conclusions:**

The intervention was acceptable to participants with most receiving and accepting the required number of visits. Key strenghts were high staff retention and fidelity to the intervention. This community-based intervention was an acceptable and feasible approach to reduce virological failure among children living with HIV.

**Trial registration:**

The ZENITH trial was registered on 25 October 2012 in the Pan African Clinical Trials Registry under the trial registration number PACTR201212000442288. It can be found at http://www.pactr.org/ATMWeb/appmanager/atm/atmregistry?dar=true&tNo=PACTR201212000442288.

**Electronic supplementary material:**

The online version of this article (10.1186/s13012-018-0762-5) contains supplementary material, which is available to authorized users.

## Background

Sustained adherence to antiretroviral therapy (ART) is critical to achieving optimum long-term clinical outcomes. Compared to adults, children exhibit lower ART adherence resulting in poorer rates of viral suppression [1–3]. Children younger than 15 years comprise 7% of all people living with HIV but 13% of all HIV-related deaths, due to lower levels of both ART coverage and viral suppression in children compared to adults [4]. This group should thus be prioritised for interventions to improve clinical outcomes [5].

We conducted a randomised controlled trial in Zimbabwe of a community-based support intervention aimed at reducing virological failure and improving retention in care in children aged 6–15 years (the ZENITH trial) [6]. We recruited children newly diagnosed with HIV from seven communities in Harare and allocated them 1:1 to receive supportive home visits from community health workers (CHWs) in addition to clinic-based care or clinic-based care only (standard HIV care). The primary outcomes were proportion of children on ART with an HIV-1 viral load > 400 copies/ml 12 months post-initiation, and proportion missing two or more routine appointments by the end of follow up (18 months). Secondary outcomes included self-reported adherence, all-cause mortality, number of unscheduled visits to primary health clinics (PHCs), number of hospital admissions, proportion of children who changed to second line ART as a result of treatment failure, and a composite outcome of mortality, treatment failure, non-initiation of ART, and loss to follow-up. As detailed elsewhere, children in the intervention arm had significantly reduced treatment failure at 12 months post ART initiation (33.0 versus 48.2%, adjusted odds ratio 0.47, 95% CI 0.24–0.91, *p* = 0.03) and a significantly lower proportion had the composite outcome (43.8% vs 58.0%; adjusted odds ratio 0.50; 95% CI 0.28–0.89; *p* = 0.02) [6]. No differences between arms were identified for the remaining outcomes. This was the first randomised controlled trial to demonstrate improved virological outcomes among children receiving community-based support.

We conducted a process evaluation to assess the intervention’s fidelity, acceptability to CHWs and participants, and feasibility of implementation. For the purposes of this process evaluation we have defined acceptability of the intervention as study participants’ willingness to receive CHW visits (at home or other community location) as well as CHW retention throughout the intervention. In this paper, we report on the design and delivery of our community-based intervention, highlighting its strengths and weaknesses to identify lessons that could prove useful for future adaptation or scale-up.

## Methods

### Intervention design and conceptual framework

Children’s access to and sustained engagement with health services heavily depends on adult caregivers [7], who may not be their biological parents and may be living with HIV themselves [8]. There is a wealth of literature describing caregivers’ own support needs in caring for children living with HIV. Caregivers often report feeling ill-equipped to cope effectively, describing challenges including social isolation, AIDS-related stigma and discrimination, and competing family demands [9–12].

The importance of providing social support to caregivers of children living with HIV for adherence to treatment is also well documented [13, 14]. Two facilitators of adherence and improved treatment outcomes for young children are follow-up counselling for caregivers to provide further information, and disclosure of HIV status to children and family members [15, 16]. For example, a multicentre cohort study in South Africa showed that home visits by lay ‘patient advocates’ to the households of newly diagnosed children was associated with improved virological suppression over 4 years [17]. These patient advocates were trained to provide adherence counselling and to address psychosocial problems within households.

Our formative research showed that caregivers of children living with HIV confronted many of the same barriers in managing children’s care as adults responsible for their own treatment [12]. Since home-based support has been shown to improve retention in care, adherence to ART, and viral suppression among adults in sub-Saharan Africa [13, 18], we hypothesised that a similar approach would prove effective for children.

**Fig. 1:**
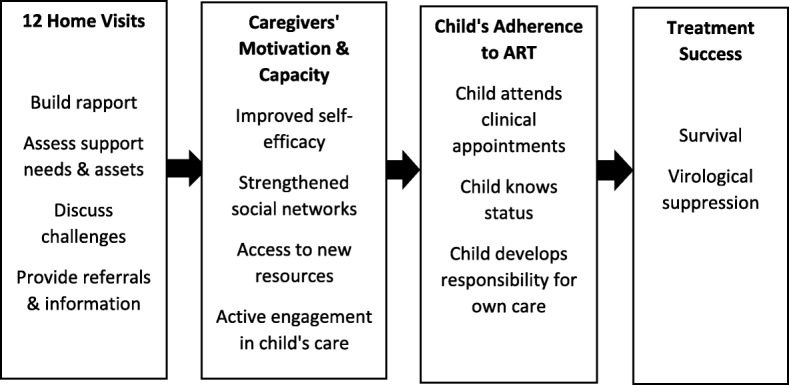
Theory-driven conceptual framework of intervention effect

Figure 1 presents our theory-driven conceptual framework for how intervention activities may have contributed to improved clinical treatment outcomes. Our intervention is rooted in social cognitive theory, with particular focus on the concept of “self-efficacy”, which relates to an individual’s ability to act on his or her intentions [19]. We adapted the structure and content of the CHW home visits on an existing strengths-based counselling programme for linking ART patients to services which is a CDC-approved Best Practice intervention [20]. This approach to case management works to build self-efficacy through a facilitated process of identifying internal strengths, social support, and problem-solving skills. Table 1 summarises the delivery and content of home visits. The intervention addressed social support and other known determinants of children’s treatment success such as knowledge of their HIV status and consequent ability to take increasing responsibility for their own medical appointments, prescription refills, and adherence [21–23]. We conceptualised that structured home visits to children together with their caregivers would help build up motivation and self-efficacy, which would make it more likely that caregivers would follow up on care and feel less helpless [24, 25]. Referrals to support organisations within communities would also support families dealing with poverty, which poses competing demands on caregivers’ time, resources, and energy. Food insufficiency and hunger have previously been identified as drivers of non-adherence while studies in Uganda and Zambia have shown that food supplementation may be associated with improved HIV treatment outcomes [26–28]. Referrals to community-based support groups for adolescents would help build up children’s own skills for self-care, as well as tackling their psychological distress, isolation, and fears of stigma [11, 29].

**Table 1:** Summary of home visits schedule and session content

Visit type	When?	Key content
1. Initial visit	Within 1 week from enrolment	• Introduction to home visits
2. Introduction	1 week from first clinical visit	• Information and resources on HIV and treatment • Family mapping • Assessment of disclosure to child/others
3. Planning for successful treatment	Within 2 weeks after the first treatment monitoring clinic appointment NB: only for ART-eligible participants	• Development of a personal treatment plan • Assessment of need/eligibility for referrals to locally available services • Discussion of managing drug stock-outs/additional treatment charges
4. Side effects	1 month later NB: only for ART-eligible participants	• Discussion around side effects and treatment experience to date • Provision of information on managing side effects
5. Disclosure	1 month later	• Discussion around disclosure to the child
6. Maintenance	3 months later	• Discussion around long-term maintenance of treatment
7. Ongoing support	Every 3 months	• Follow-up on issues emerging from clinical monitoring appointments • Review of need for referrals, assistance with disclosure, and/or support for testing of other household members • Answering questions, facilitating identification of solutions to emerging challenges, and providing relevant information
8. Unscheduled	At the discretion of the CHW or after a request by the study nurse	• Case by case

NB: once child becomes ART eligible, visits 2–4 are repeated with focus on ART-specific activities

We therefore designed the ZENITH intervention to offer community-based support through home visits to the households of children (6–15 years) newly diagnosed with HIV. Children enrolled in the trial were individually randomised to receive the intervention (12–15 home visits) as well as standard HIV care at the clinic or standard HIV care at the clinic only. Home visits were conducted by a pre-existing cadre of CHWs, trained to facilitate structured discussions at critical points in a child’s progression through HIV diagnosis, treatment initiation, and long-term maintenance for 72 weeks post HIV diagnosis. Detail on recruitment and experiences of the CHWs who took part in this study is published elsewhere [30].

Children who transferred out of study communities were discontinued from receiving the intervention, although efforts were made to ensure they were enrolled in clinical care at their new residential location.

### Evaluation methodology

To evaluate intervention fidelity, acceptability, and feasibility, we used field manuals kept by each CHW, records from monthly supervisory meetings, and participant data collected by study nurses throughout the trial.

The CHW field manual was a tool to guide CHWs’ work and document their experiences (Additional file 1). It described the objectives and procedures for each visit and provided space for the CHWs to record key activities for each visit. Notes in field manuals were translated and transcribed from the local language Shona to English and entered into an Excel spreadsheet. Quantitative data extracted from all field manuals included visit date, success in meeting the caregivers and their relationship to the child, duration of visit, and visit type, analysed using Stata 14.0 (StataCorp). The CHW manual provided guidance on activities conducted during each visit. The number and type of activities differed depending on the visit type. CHWs were asked to check activity tick boxes after each visit indicating the topics and activities they were able to complete. Where CHWs had not checked tick boxes a research assistant retrospectively completed them based on the CHW’s visit notes.

Minutes from monthly supervisory meetings recorded by the field manager were used to capture CHW attendance and retention. Project records detailed training content and coverage.

We used participant data collected through end line trial questionnaires to ascertain participant outcomes such as transfers out from the study catchment area or death and disclosure of HIV status. This data was extracted from the trial database and analysed using Stata 14.0 (StataCorp, TX, USA).

## Results

### CHW recruitment, training, and retention

CHWs were recruited from the study communities and trained to offer treatment literacy, counselling, practical advice for emerging challenges, and links to appropriate referrals. Twenty CHWs (3 male, 17 female) were selected from a pre-existing pool of volunteers for the Child Protection Society (CPS) whose primary function is to provide support for vulnerable children in Zimbabwe [31]. Eligibility criteria included residence in the local community for at least 5 years, functional literacy, willingness to travel on foot between households and to regularly visit the clinic, as well as experience of nurturing others. The CHW age range was 29–58 years. All had at least secondary education, with several having achieved a tertiary level qualification, although this was not a requirement. Phone airtime allowance was provided to CHWs for communication with participants when pre-booking/scheduling visits as well as a monthly allowance of US$20.

Training was conducted over 2 weeks followed by 2 weeks of intense on-the-job supervision. The training focused on basic information related to HIV transmission, prevention, treatment regimens, care needs, and orientation on common challenges families face in initiating treatment and sustaining adherence and retention in care. Key aspects of training included encouraging HIV testing of other family members and facilitating discussion around age appropriate disclosure of HIV status to the child and to other family and community members, where feasible. Refresher training was conducted after 1 year.

Monthly meetings with the field manager were carried out to share experiences, build team unity, address any difficulties emerging during the program, and provide CHWs with up-to-date information and guidance on treatment. At each meeting, CHWs gave patient specific progress updates to identify emerging challenges. Average attendance at monthly debrief meetings was 16 CHWs while 17 CHWs were present at the refresher training. One CHW relocated during the first year of the study. The remaining 19 CHWs were still working for ZENITH at the end of the trial. The median number of participants allocated per CHW was 9 (range 5–15).

### Intervention delivery: frequency of visits

**Fig. 2:**
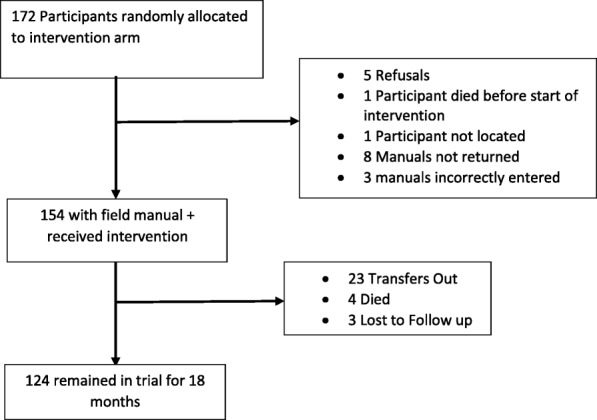
Participant intervention cascade

Out of 334 children recruited into the trial, 166 were randomised to receive the intervention. An additional 6 children received the intervention because another child in the same household had already been randomised to the intervention arm, giving a total of 172. Five (2.9%) households refused to have home visits after randomisation, one child could not be located and one child died before receiving any visits. Eight field manuals were not returned and three were incorrectly entered (Fig. 2).

**Fig. 3:**
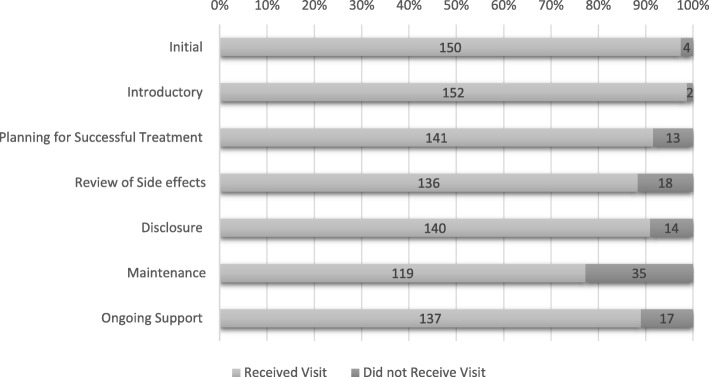
Proportion of participants to have at least one successful visit by visit type (*N* = 154)

Among the 154 children who received at least one home visit and had a completed manual, 1553 home visits were successfully conducted, i.e. contact was made with the family (median 11 per participant, range 1–20), of which 108/1553 visits (7.0%) were unscheduled. In addition, for all attempted visits, 85/1664 (5.1%) scheduled and 26/134 (19.4%) unscheduled visits were conducted in which CHWs were not able to make contact with the family. Thirteen (8.4%) participants received 5 visits or fewer; 10 because they transferred out of Harare and 3 because they died. Before the end of the trial, 23/154 (14.9%) participants with a field manual transferred out of the trial clinics while 4 (2.6%) died and 3 (1.9%) were lost to follow-up (Fig. 2). The initial and introductory visits had the highest coverage with over 95% of participants receiving these visits while the treatment maintenance visit had the lowest coverage with only 77% of participants receiving this visit (Fig. 3).

The length of visits ranged from 9 to 150 min (mean length 42 min). The shortest visit on average was the initial (first) visit (mean 36 min) while the longest was the introductory (second) visit (mean 48 min). The most commonly recorded reason for an unscheduled visit was non-adherence, i.e. the nurse reported the child was not going to the clinic or not collecting their medication (*n* = 42). Other reasons included repeat visits to two families that did not accept the diagnosis (*n* = 5), following up for changes of address (*n* = 9), at the caregiver’s request (*n* = 5), or the child being unwell (*n* = 5).

CHWs recorded which family members they met at each visit, although on 517/1553 (33.3%) occasions they recorded only the number of people rather than their identities/relationship to the child. The child was recorded to have been present at 388/1553 (25.0%) visits with 137/1553 (8.8%) visits recorded that only the child was present. Three children (aged 13, 15, and 15 at enrolment) were defined as their own primary caregiver because they usually met the CHW alone. The child’s mother (*n* = 658), grandmother (*n* = 216), and aunt (*n* = 272) were the caregivers seen by CHWs most frequently. The field manager accompanied each CHW on up to 4 visits as a quality assurance measure and to provide additional support when required. While the intention was for visits to take place at home, 15/154 (9.7%) families requested at least one visit elsewhere (at the clinic, in the park, at their workplace, or at the shops).

### Intervention delivery: content of visits

CHWs fully completed the activity tick boxes for 585/1428 (41.0%) visits. For 725/1428 (50.8%) and 118/1428 (8.3%) visits, the CHWs did not complete the tick boxes or partially completed them, respectively. For these 843 visits, a research assistant retrospectively completed the tick boxes using CHWs’ visit notes. In some instances, there was no information on whether or not an activity had been completed.

As shown in Additional file 1, activities varied per visit and included providing information and resources on HIV and treatment and assisting with disclosure and post-disclosure discussions, following up on issues emerging from clinical monitoring appointments, providing information on locally available services such as support groups and making referrals to these based on each family’s need, and encouraging HIV testing of other family members.

We hypothesised that understanding family structures would be important in creating rapport between CHWs and their clients and would enable CHWs to help their clients identify support available to them. CHWs reported completing the family mapping exercise with 135/153 (88.2%) participants during the introductory visit. The exercise revealed potential sources of support such as neighbours and school teachers.

CHWs discussed disclosure with the child in 149/167 (89.2%) of visits dedicated to disclosure. During the “planning for successful treatment” visit, CHWs completed assessment of need for referrals and following up on them in 73/141 (51.8%) visits. In subsequent visits, CHWs reported following up on previously made referrals in 265/559 (47.4%) visits. However, in 110/559 (19.7%) visits, there was no information on whether follow-up was done, while in 184/559 (32.9%) visits this task was indicated as not applicable. Referrals were made most commonly to support groups, food supplementation programmes, organisations that helped secure school fees, and assistance in obtaining birth certificates (required for qualifying for other social and educational services). CHWs also helped caregivers navigate bureaucracy, for example, obtaining an affidavit or completing income generating project funding proposal form. Following the trial, 67/90 (74.4%) of participants reported that they had knowledge about support groups and 31/90 (34.4%) children in the intervention arm reported having joined a community-based support group. The CHW supervisor also had some discretionary funds that were occasionally used to pay for school fees or buy food and purchase school uniforms for participants.

During support visits, CHWs were tasked with reviewing participants’ personal treatment plans and ongoing treatment. CHW manuals showed that this was done in 477/559 (85.3%) visits, with no information recorded in 63/559 (11.3%) visits. CHWs were also instructed to answer any questions the clients might have from their clinical appointments regarding HIV, its treatment and monitoring. They reported having done this in 77/153 (50.3%) introductory visits, with no information as to whether or not it was done in 75/153 (49.0%) of the manuals.

At baseline, 79/154 (51.3%) of children knew their HIV status and 41/154 (26.7%) learnt their status during the intervention period. Among study participants, 19/139 (13.7%) children never learnt their HIV status. The majority of children older than 10 years at baseline (80/82; 97.6%) were told their HIV status while only 5/26 (19.2%) of those younger than 8 years were told their HIV status. Relatives other than the caregiver were aware of the child’s status for 97/154 (63.0%) participants while friends, church pastors, and school headmasters were reportedly informed in only 6.5, 5.8, and 1.9% of participants, respectively. At baseline, 64/154 (41.6%) of mothers were either dead or unreachable while 71/154 (46.1%) were known HIV positive. During follow-up, 6/19 (31.6%) eligible mothers were tested. Among fathers, 71/154 (46.1%) were dead or unreachable at baseline and 41/154 (26.6%) were HIV positive. Only 4/42 (9.5%) eligible fathers were tested for HIV during follow-up; 30/42 (71.4%) fathers did not test, and there were no data for the remaining 8. Among study participants, 47/154 (30.5%) reported having a sibling tested during the study.

## Discussion

### Acceptability

Participants found the intervention acceptable. Of the 470 eligible families approached for the study, only 25 (5.3%) refused consent, for reasons which are unknown but may have been because they did not want home visits [30]. Additionally, 5 families (2.9%) refused home visits after signing the consent form. Although the intervention was tailored to be delivered at home, it was noted that some families and participants (15/154; 9.7%) opted to meet with the CHWs in other locations such as the clinic, at their workplaces, or in the park. In most instances these participants were not comfortable with home visits as they feared accidental disclosure to other family members and neighbours; however, other reasons included convenience such as the preferred meeting location being the local market where caregivers worked. In some (4/15; 26.7%) cases, the family later invited the CHW to their home, having established a relationship of trust.

The intervention was also acceptable for CHWs to deliver, with almost all of them (19/20) retained at the end of the intervention. This may be due to the extensive training and the ongoing mentorship and support through on-the-job supervision and monthly meetings [30].

Qualitative work conducted as part of the trial (detailed elsewhere [12, 16, 30, 32]) including in-depth interviews with caregivers and the CHWs showed that HIV-related stigma and discrimination was one of the key barriers faced and overcame in the delivery of this intervention [30, 32].

The proportion of participants who received each visit type remained constant above 85% for all visit types, with the exception of the maintenance visit where 77% of participants received that visit. This is indicative also of acceptability for study participants where no refusals/drop outs occurred after randomisation.

### Fidelity

The median number of visits was 11 per participant out of the stipulated 12–15 visits. However, this includes data from participants who exited the study before the end of the intervention period. CHWs conducted a high proportion of the stipulated activities at each visit, such as providing treatment support, facilitating disclosure, and making referrals to community-based support services. CHWs discussed disclosure with the child/family in over 89% of cases and assisted their clients with developing and reviewing their personal treatment plan in over 85% of participants. We attribute this to the clear and structured design of the manual that was tailored to guide the CHWs through each visit and activity and in-depth training provided (Additional file 1).

Fidelity to the planned activities was good overall as a review of the content of CHWs’ visits showed that in providing HIV treatment support to households of children living with HIV, CHWs also assisted with bureaucratic issues such as the need for assistance to access services available for them like obtaining documentation for children, such as birth certificates. The CHW intervention was flexible in that CHWs were able to provide referrals on the basis of each family’s need which varied for each household. Our intervention also showed there is a need for additional resources and appropriate referrals to organisations that can provide support in the form of food supplementation and resources to support school attendance [33].

### Feasibility

Only 5% of scheduled visits did not occur because the CHW could not make contact with the participants and caregivers. We did observe a high rate of transfers among study participants to areas that were not within the study catchment area (23/154; 15% of participants). Other studies have reported unstable caregiving arrangements for children with HIV, with frequent change of caregiver and movement between households, which is associated with poor treatment outcomes [34, 35]. This can also hinder delivery of support interventions, as was the case in our study where CHWs could not continue to offer support to participants who had transferred outside the area.

While the CHWs managed to deliver a median of 11 visits per participant, such an intense model may not be scalable for large populations and national programs due to labour intensity. Adaptations of this model such as delivery of fewer visits targeting critical periods in the HIV care cascade, for example, post-diagnosis, around ART initiation, and so on, should be evaluated. We observed that study nurses communicated regularly with CHWs. When participants missed clinic appointments, the nurse contacted the CHW to request follow-up which often resulted in an unscheduled visit (134/1564; 8.6% of all visits). This may have resulted in timely address of issues affecting retention in care and adherence to treatment, and contributed to the significantly higher HIV virological suppression observed in the intervention arm. Ensuring strong links and regular communication between clinic staff and community support workers will be important additions to support adherence and retention in care, particularly in children.

Based on the findings of this evaluation, we believe that the ZENITH intervention provided at critical time points in the HIV care cascade for older children and adolescent may be an effective and sustainable tool to improve HIV treatment outcomes in older children and adolescents. However, the intervention will need to be tailored to context. For example, this intervention was delivered in an urban setting and may require adaptation for implementation in rural settings where CHWs may have to travel longer distances in difficult terrain [36]. This is likely to have implications on the number of visits each CHW can conduct each day, the total number of successful visits each CHW can make, and the time spent travelling to each participant household by CHWs. Adaptations may include provision of bicycles for CHWs to move between households or conducting the visits at the local healthcare facility on routine appointment dates to allow for ease of access. The CHWs who worked on this study lived in the study communities and could travel mostly by foot for their visits. Access to HIV treatment services and health care delivery challenges have also been shown to be different between people living with HIV in rural versus urban settings, and these differences may have a bearing on the feasibility of delivering this intervention in rural communities [37, 38]. Fewer visits could help reduce costs without compromising effectiveness.

### Strengths and limitations

A strength of our process evaluation was that we were able to use data from multiple sources collected throughout the trial. A limitation was that not all CHW completed their manuals fully. One of the indicators we report in this paper, activities done on each visit, had to be retrospectively compiled using visit notes as CHWs did not always tick the list of activities they had completed in the manual. This may have underestimated the proportion of activities that the CHWs undertook.

An additional study limitation was that we could not definitively ascertain to whom the intervention was delivered. On 517 (41.0% of visits) occasions, the CHW reported only the number of people present at each visit, and in some instances where the person is named, we were not able to ascertain their relationship to the child. As the details of the caregiver the CHWs met on visits was not accurately recorded, we are unable to determine if may have resulted in difficulties in delivering the intervention (no contact visits/rescheduled visits/more frequent unscheduled visits).

While we are unable to definitively ascertain the exact component(s) of the intervention or the event pathway that led to the improved viral suppression observed among intervention arm participants, we believe that our holistic approach provided multi-component support that created an enabling environment for positive treatment outcomes (Fig. 1). Disclosure of HIV status is a facilitator for retention in care [39] and was therefore included as part of the intervention, whereby CHWs were trained to address disclosure issues. By the end of the follow-up period, 78% of children in this study were aware of their status. Disclosure of HIV status to the child, immediate family, and relatives may have been a key driver of the intervention’s effect. Similarly, social protection through the use of unconditional cash transfers and psychosocial support have been shown to reduce HIV risk behaviour among adolescents in South Africa and may also be effective in improving treatment outcomes [40]. In this intervention, social protection was provided through psychosocial support together with assistance in navigating government systems for obtaining birth certificates and school registration, and through other service-provision organisations. This also may have been on the pathway to the intervention effect observed.

## Conclusion

This CHW intervention was an acceptable and feasible approach to support households of children living with HIV. Key strengths of our intervention were high staff retention and high fidelity. These findings have potential for scalability particularly in African settings where community health workers are a pre-existing cadre of health workers and the burden of HIV is high. The generalisability of these findings is limited to an urban setting, and there is a need for further investigation of the effect of the intervention in other settings; particularly rural settings where community structures and norms may alter the feasibility of such an approach. Adaptations of this model to incorporate fewer, targeted visits must also be evaluated. Future studies should prioritise cost analysis as these are critical in informing the feasibility of scale up and subsequent incorporation of these interventions in national health policies.

## Additional file

Additional file 1: Voluntary community health worker field manual. (DOCX 304 kb)
