# Supplementary material for: Community health worker support to improve HIV treatment outcomes for older children and adolescents in Zimbabwe: a process evaluation of the ZENITH trial
Source: Implement Sci. 2018 May 23;13:70. doi: 10.1186/s13012-018-0762-5 (PMC5966852; doi:10.1186/s13012-018-0762-5)
Supplement: Supplementary file 1 — Voluntary community health worker field manual. (DOCX 304 kb) [file 13012_2018_762_MOESM1_ESM.docx]

|  | 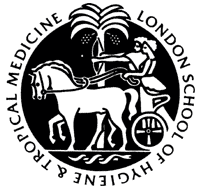 |
| --- | --- |

**ZENITH**

**Zimbabwe study for Enhancing Testing and Improving Treatment of HIV in Children**

Field Manual & Case Record

ZENITH is a randomised controlled trial to test whether support to households of HIV-infected children improves ability of children to keep attending their clinical appointments (retention in care) and their ability to take HIV medication regularly and as prescribed (adherence). This community-based support intervention, consisting of structured support visits to households of HIV-positive children, will be delivered by volunteer lay workers (VLWs) already working in the study communities as part of the Child Protection Society. The trial will start recruiting clients in February 2013 and the intervention will be delivered to each client for two years.

## ZENITH is a joint collaboration between:

The Biomedical Research and Training Institute (BRTI), Harare, Zimbabwe

Harare City Health Services, Zimbabwe

The Child Protection Society, Zimbabwe

London School of Hygiene and Tropical Medicine (LSHTM), UK

**TABLE OF CONTENTS**

[1. The ZENITH trial 1](#_Toc348679423)

[1.1 Why is this Trial being Conducted 1](#_Toc348679424)

[1.2 Description of the Intervention 1](#_Toc348679425)

[1.3 Timing & Content of Visits 2](#_Toc348679426)

[1.4 Schedule of Clinic Follow-up of Clients 4](#_Toc348679427)

[1.5 Summary of Visits for clients who start ART at recruitment 5](#_Toc348679428)

[1.6 Summary of Visits for clients ineligible for ART at recruitment 6](#_Toc348679429)

[1.7 What is in this Manual & Case Record 7](#_Toc348679430)

[2. General instructions 8](#_Toc348679431)

[3. Client Details 10](#_Toc348679432)

[4. Record of Home Visits 11](#_Toc348679433)

[5. Record of Individual Visits 13](#_Toc348679434)

[INITIAL VISIT 14](#_Toc348679435)

[INTRODUCTORY VISIT 16](#_Toc348679436)

[PLANNING FOR SUCCESSFUL TREATMENT 18](#_Toc348679437)

[REVIEW OF SIDE EFFECTS 20](#_Toc348679438)

[DISCLOSURE 22](#_Toc348679439)

[MAINTENANCE VISITS 25](#_Toc348679440)

[MAINTENANCE (on ART) 26](#_Toc348679441)

[DISCLOSURE (not on ART) 28](#_Toc348679442)

[ONGOING SUPPORT (on ART) 30](#_Toc348679443)

[ONGOING SUPPORT (not on ART) 42](#_Toc348679449)

[UNSCHEDULED VISIT 58](#_Toc348679457)

[6. Additional Information 60](#_Toc348679459)

[6.1 COMMUNITY BASED ORGANISATIONS (CBOs) AND SUPORT GROUPS IN STUDY SUBURBS 61](#_Toc348679460)

[6.2 KEY OBJECTIVES OF HOME VISITS 62](#_Toc348679461)

[6.3 KEY HOME VISIT SKILLS 63](#_Toc348679462)

[6.4 HELPFUL TIPS ON FACILITATING DISCLOSURE 64](#_Toc348679463)

[6.5 HELPING CAREGIVERS IDENTIFY THEIR STRENGTHS AND SKILLS AND SUPPORT NETWORKS 65](#_Toc348679464)

[6.6 FAMILY MAPPING (Resource 1) 67](#_Toc348679465)

[6.7 STENGTHS & SKILLS (Resource 2) 68](#_Toc348679466)

[6.8 Tips for Positive Living 69](#_Toc348679467)

[6.9 Developing a Personal Treatment Plan for Children 70](#_Toc348679470)

# 1. The ZENITH trial

The trial will be conducted in six suburbs in southwest Harare. As part of ZENITH, HIV testing will be offered to *all* children aged 6-15 years who attend the polyclinics in the six study suburbs, and decentralised HIV care (i.e. HIV care provided at the primary care clinics) will be provided at all the respective polyclinics.

Children who test HIV-positive will be invited to enrol in the ZENITH Trial and trial participants will be randomly selected to receive the intervention (home visits) as well as standard HIV care at the clinics. Those who are not selected to receive the intervention will receive standard HIV care at the clinics only.

The intervention will centre around a standardised set of the home visits, delivered at critical points in a child’s progression through HIV diagnosis, treatment initiation, and long-term maintenance, including adherence to prescribed drug regimens.

## 1.1 Why is this Trial being Conducted

Older HIV-infected children have a disproportionately high risk of poor outcomes, including retention in care and adherence, compared to adults.

Suboptimal care-giving is among the potential factors as treatment outcomes for chronic childhood illnesses are greatly influenced by caregivers’ awareness and willingness to invest time and effort into accessing care. Caregivers struggle to cope with children’s demands for knowledge and psychosocial support, and addressing disclosure to the child and to others. This may be compounded by pressures within the family, including poverty, loss of other family members due to HIV, and isolation as well as discrimination by others in the community as a result of the “presence of HIV” within the household. Furthermore, service provision for children living with HIV may also not match their needs. Studies in Africa demonstrate a “disconnect” between what is provided to HIV-positive children and what they state they need.

The intervention is designed to support children and their primary caregivers so that they remain engaged with HIV care services. It adopts a “family centred approach,” recognising that children’s retention in care depends on not only on their own understanding and acceptance of their condition and personal motivation, but also on their specific family circumstances. These include, but are not limited to, relationship to a primary caregiver, HIV status of others living in the household, levels of open communication and support within and beyond the family, and access to resources (financial, instrumental and psychosocial). These factors will differ between newly diagnosed children living with HIV, and are likely to change over time, particularly with increased age of the child and at different stages of HIV treatment.

## 1.2 Description of the Intervention

There are points at which patients may require more intensive support and assistance to prevent loss to follow up. These are:

1. Diagnosis with HIV, when patients require reassurance, information on next steps, and help accepting their status;
2. Enrolment in ART, when they need to learn the requirements of their regimen, what to expect, and how to manage side effects;
3. Establishing long-term treatment maintenance, when the importance of adherence needs to be understood, as well as other aspects of living positively; and
4. Changes to treatment (or demonstrated poor adherence/appointment attendance), when patients may require help in re-committing to the treatment process and support to maintain adequate levels of motivation.

The experience of children, however, differs from that of adults, as they are directly dependent on their caregivers, particularly at younger ages. Their retention in care and adherence to treatment will depend on parents, extended family members, and potentially others in their community, all of whom need to be explicitly targeted by efforts to optimise treatment success in their children.

The aim of this intervention, therefore, is to actively engage caregivers in identifying feasible ways to ensure the child’s retention in care, and addressing potential barriers they may face, and supporting these measures by providing appropriate referrals to locally available services.

The community based intervention in ZENITH will centre around 12-15 home visits (depending on when the child becomes eligible for ART), conducted over a two-year period, as described below. The first 5-8 visits will be structured around a series of standardised activities, followed by a further 7 visits that are designed to be significantly shorter and less formal, unless particular concerns have been identified.

Home visits will be based on a *strength-based case management approach*, Strength-based case management focuses on people’s skills, resources, and positive experiences in the face of adversity, rather than on constraints. Facilitated discussions thus lead participants through a process of identifying their own strengths, planning for successful adherence, and developing practical solutions as challenges emerge. Where possible, referrals to available services in the area are offered, with practical assistance in following these up (such as contacting a local support group or checking eligibility for a food supplementation programme).

The home visits will also be linked to clinical care, with regular meetings between nurses, counsellors, the project physician, and home visitors held to discuss patients and share information that may need to be addressed at either clinical monitoring appointments or within the community setting.

## 1.3 Timing & Content of Visits

**1.3.1 Introductory visit after enrolment into Trial:** As soon as a child is enrolled into the intervention arm of the trial, a VLW will be allocated to the child and his/her household. The VLW will make a short visit to the household before the child has their first assessment appointment at the clinic. The main purpose of this visit is for the VLW to confirm the child’s address and to introduce him/herself to the child and caregiver.

**1.3.2 Children who start ART** will receive counselling (along with their primary caregiver) and start on ART within 2 weeks of their clinical review. They will then be requested to return for their next clinical appointment after a further 2 weeks, and subsequently for two more appointments at 4 week intervals, after which they will be monitored every 3-months. Thus, children enrolled in ART will visit the clinic twice in the first month, twice more in the next two months, and then 7 more times over the two-year follow-up period.

Home visits will be integrated into this clinical schedule, with community-based follow-up occurring between each scheduled clinic appointment. The first 5 visits will occur with greater frequency, and consist of a set of standardised, structured activities. These will be followed by 7 additional, shorter, and more informal visits. Children who are enrolled immediately in treatment will receive a total of 12 structured home visits over two years. Table 1 describes the schedule and content of home visits.

**1.3.3 Children who are not yet eligible to initiate ART** will be monitored at 3-monthly intervals, and enrolled in treatment according to national guidelines. However, they will receive home visits in the same way as children on treatment, although each visit will be shorter and focus on the broader issues related to living with HIV, with just a brief overview of what they might eventually expect during treatment. The importance of maintaining their regular clinic appointments to monitor their condition will be emphasised, and referrals made to other organisations in the area offering relevant services such as support groups, food supplementation or assistance with travel to appointments. Once the child initiates treatment, s/he will receive the ART-focused structured activities usually provided in the first 3 visits. Thus children enrolled in treatment later than the time of initial diagnosis will receive up to 15 home visits over two years.

Below is the visit schedule which the Volunteer lay worker will follow and the activities expected to be done at each visit. Feedback on problems, progress of the child will be given to the research nurse/ research assistant at the respective clinic. Regular meetings between VLWs and the Project will be held to discuss any problems encountered during visits.

## 1.4 Schedule of Clinic Follow-up of Clients

**RECRUITMENT: VLW Trial**

**WEEK 0**

**Initial Assessment**

**1 MONTLY VISIT**

- Week 8
- Week 12

**3 MONTLY VISITS***

- Week 24
- Week 36
- Week 48
- Week 60
- Week 72
- Week 84
- Week 96

*All participants must complete 96 weeks of follow-up.

**WEEK 4**

(2 week post ART)

**Routine follow-up**

**ART Eligible**

ART counselling & Start ART

**WEEK 4**

**Routine follow-up**

**ART Ineligible**

**WEEK 2**

**ART Eligibility**

**Visit 1:**

“Check visit”

**Visit 2:**

Following initial assessment

**Visit 3:**

2 weeks after starting ART

**Visit 3:**

Following ART eligibility visit

**Visits 4-5:**

Following each 1 monthly visit

**Visits 6-12:**

Following each 3 monthly visit

**If participants start ART during the follow-up period:**

- Book an additional visit at 2 & 6 weeks after starting ART
- Then revert back to the usual visit schedule

2 additional home visits following the 2 and 6 week post-ART appointment

**Visits 4 onwards:**

Following each 3 monthly clinic visit

## 1.5 Summary of Visits for clients who start ART at recruitment

| **Visit Name** | **When?** | **Content** |
| --- | --- | --- |
| **1. Initial visit** | Between enrolment and Initial assessment | - Confirm address - Introduce yourself to client and caregiver - Introduction to home visits |
| **2. Introduction** | Within a week of initial assessment | - Answering questions from clinical appointments - Provision of information & resources on HIV and treatment - Family Mapping - Identification of strengths & resources available to client & household - Assessing disclosure to child/others - Assessing need for family testing |
| **3. Planning for successful treatment** | Within the next 2 weeks, following the first treatment monitoring clinic appointment for those who have initiated ART | - Discussion of treatment experience to date - Development of a Personal Treatment Plan - Assessment of need/eligibility for referrals to locally available services - Answering questions from clinical appointments - Discussion of managing drug stock-outs/additional treatment charges - Follow-up on disclosure to child/others - Follow-up on testing uptake by other household members |
| **4. Side Effects** | One month later (within 1 week of the next clinical appointment for those on ART, to follow-up any issues identified by clinic staff) | - Review of the Personal Treatment Plan - Follow-up on referrals made at previous visit - Facilitated discussion around side effects and treatment experience to date - Provision of information on managing side effects - Answering questions from clinical appointments - Follow-up on disclosure to child/others - Follow-up on testing uptake by other household members |
| **5. Disclosure** | One month later, after 2^nd^ monthly follow-up appointment for those on treatment, and after 1^st^ 3-monthly assessment for those not yet eligible  *NB: Once child becomes eligible, Visits 3-4 are repeated, with focus on ART-specific activities* | - Review of the Personal Treatment Plan - Facilitated discussion around disclosure to the child - Answering questions from clinical appointments - Follow-up on referrals - Follow-up on testing uptake by other household members |
| **6. Maintenance** | Three months later | - Review of the Personal Treatment Plan - Check on disclosure to the child - Facilitated discussion around long-term maintenance of treatment - Answering questions from clinical appointments - Follow-up on referrals - Follow-up on testing uptake by other household members |
| **7. Ongoing Support** | Every 3 months | - Follow-up on issues emerging from clinical monitoring appointments - Review of Personal Treatment Plan - Review of need for referrals, assistance with disclosure, and/or support for testing of other household members - Answering questions, facilitating identification of solutions to emerging challenges, and providing relevant information |

## 1.6 Summary of Visits for clients ineligible for ART at recruitment

| **Visit Name** | **When?** | **Content** |
| --- | --- | --- |
| **1. Initial visit** | Between enrolment and Initial assessment | - Confirm address - Introduce yourself to client and caregiver - Introduction to home visits |
| **2: Introduction** | Within a week of initial assessment | - Answering questions from clinical appointments - Provision of information & resources on HIV - Family Mapping - Identification of strengths and resources available to client and household - Assessing disclosure to child/others - Assessing need for family testing |
| **Home visits continue to follow the schedule of clinic visits.**  **Once a child becomes eligible for ART, insert the 3 visits that are focused on treatment and adherence.** | | |
| **3: Disclosure** | One month later, after 1^st^ follow-up appointment  (Week 4) | - Conversation about recent monitoring appointment, including the significance of being found to be ineligible for ART. - Emphasis on positive living - Answering questions emerging from recent appointment or general - Reinforcing importance of maintaining monitoring appointments to check for eligibility and manage other health issues. - Facilitated discussion around disclosure, with suggestions (and provision of materials) for how to disclose to children at different ages; offer to provide assistance with disclosure at a subsequent visit - Assessment of need/eligibility for referrals to locally available services - Follow-up on testing uptake by other household members |
| ***Until eligibility or end of follow up*:**  **Ongoing Support** | Every 3 months | - Follow-up on issues emerging from clinical monitoring appointments - Review of need for referrals, assistance with disclosure, and/or support for testing of other household members - Emphasis on positive living - Answering questions emerging from recent appointment - Reinforcing importance of maintaining monitoring appointments to check for eligibility and manage other health issues. |
| **Visits to be inserted if/when child initiates ART** | | |
| **Planning for successful treatment** | Within 2 weeks of initiating ART | - Discussion of treatment experience to date - Development of a Personal Treatment Plan by the caregiver, drawing on strengths and resources previously identified (may need to repeat the exercise, if visit 2 occurred a long time ago) - Answering questions emerging from clinical appointments - Discussion of how to manage drug stock-outs - Follow-up on any changes in disclosure to child/others - Follow-up on testing uptake by other household members - Follow-up on referrals previously made/taken up |
| **Side Effects** | Within 1 week of the 1^st^ monthly ART monitoring appointment | - Review of the Personal Treatment Plan, with discussion for how well it works and what changes are required - Facilitated discussion around side effects and treatment experience to date - Provision of information on managing side effects - Answering questions from clinical appointments - Follow-up on any changes in disclosure to child/others - Follow-up on testing uptake by other household members |
| **Maintenance** | Within 1 week of the 2^nd^ monthly ART monitoring appointment | - Review of the Personal Treatment Plan - Check on disclosure to the child - Facilitated discussion around long-term maintenance of treatment - Answering questions from clinical appointments - Follow-up on referrals - Follow-up on testing uptake by other household members |

## 1.7 What is in this Manual & Case Record

This manual will guide you to carry out your visits according to the study protocol, so that every child who receives visits gets the same level and type of support. It is thus very important that you follow the instructions in the manual carefully and we hope you will work with us to make this support intervention a success.

The manual gives you guidance on when you should conduct your home visits and what you should discuss with the family at each visit.

The manual also provides space for you to record details and feedback about each visit. The feedback and information from the lay volunteer workers in this record will help determine will determine if the participant is adhering to HIV care and treatment.

At the end of the manual, resources are provided to help you carry out support activities. In particular, refer to Refer to “5 Key Objectives of Home Visits” and “Key Home Visit Skills” as a general reminder. Other specific relevant resources should be referred to wherever relevant.

Information on locally available support services that clients can be referred to is included in this manual (see page 61). This will be updated from time to time.

Remember that the key to the success of the visits depends on developing a mutually trusting and respectful relationship with the client, and ideally, good rapport with everyone in the household.

# 2. General instructions

1. Note that the term “client” refers not only to the child but also their caregiver and the household of the child.
2. For each visit, sign and date the Visit Record (Page 11 of this manual) and ensure that the client or their caregiver signs the record too. Retrospective signatures will not be accepted. If a visit was arranged and you were not able to see the client- please record this and arrange another visit.
3. Please ensure that you maintain client confidentiality at all times. This will mean that you keep the case records in a secure place where no one else will have access to them. Do not discuss the client with anyone other than their healthcare provider and the relevant members of the ZENITH team and other professionals involved in the child’s care.
4. These visits are entirely voluntary. If the client does not wish to have a structured session, do not force them. However, you must record that you have made the visit and, if possible why the client did not wish to have a visit. Make sure the client knows that their usual care will not be affected if they decide not to see you for a session.
5. Contact the client to arrange a time to visit. Do not do turn up to a household unexpected.
6. A circumstance might arise whereby you may feel that an unscheduled visit is required if the client needs additional support or needs your help. If you do so, please record this visit and call it “unscheduled”. Record the details of this visit in this manual.
7. Review your case notes from previous visits before you do a home visit. Liaise with the Clinic Staff before every home visit and document relevant information or concerns to be addressed that may have arisen from the client’s clinic appointment.
8. Wherever possible, carry IEC and tools which will help you to discuss difficult issues. Pictures can be very helpful in making a child understand. The materials can be obtained through the ZENITH Office.
9. A list of community based organizations that clients can be referred to is provided in this manual (page 61). Please provide feedback you receive from the clients about these organizations to the ZENITH Team. Let the ZENITH Team know if you come across other suitable community support organisations that clients may be referred50? b/ to. This will enable clients to be referred to the best services available.
10. Refer to the resources provided at the end of this manual to help you carry out the specific support activities.
11. At the end of a visit, always remind the client of their next clinic appointment, and also tell them when you will return for the next household visit.
12. Recording notes in this manual is very important. Please ensure you write legible, detailed notes. It is better to write more rather than less. Feel free to add more sheets or to write on the sides of the sheet if the space provided is not enough. You are free to write notes in any language you prefer (English/Shona/Ndebele/other)
13. Communication with the ZENITH team about the progress of the client and the issues you face in the field is very important. Please bring your case notes at every ZENITH Meeting. The notes will help you communicate your concerns and also help to assess the needs of clients. In addition, it will help the ZENITH Team and CPS to understand the challenges you and/or the clients face, and to help identify solutions.
14. Remember to update and record changes in the client’s circumstances at every visit for e.g. guardianship, schooling, where client lives, changes in household membership etc. Record who has been told about the client’s status and update this information at every visit.
15. Complete your case record as soon as possible after the visit. If possible, avoid writing notes during the visit.
16. Allow time and opportunity for clients to ask questions or voice their concerns at every visit.
17. Be curious and don’t take everything that the client says at face value. Remember that what people say is often dictated by how they are feeling at the time. It is therefore important that you probe, without being too intrusive, so you can get to the bottom of the problem.
18. If an issue arises when you do not know the answer or have to get more information/help to assist, let the client know that you will find out. Ensure that you feedback to the client with a possible answer/solution.
19. Please be truthful and answer questions only if you know the answer. Do not try to “guess” the answer or provide information of which you are not sure, or you risk losing the client’s confidence and/or giving them incorrect advice.
20. Do not give money, food, gifts or other forms of material support to clients. This is against the rules of the support programme.
21. Do not take on more than you are able to or you will burn yourself out. Remember that it is not possible for you to solve all your client’s problems. It is important that you also look after yourself, take time to rest and relax and to speak to your colleagues for support. Remember that your colleagues may be facing the same challenges as yourself and are a valuable resource.

# 3. Client Details

*Ensure that you keep the client’s details up to date. Please record the client’s new address if they change address*

Study Number: **Z**

Client Name: __________________________________________

Date of birth (dd/mm/yyyy): //

Name of guardian: _____________________________________

Address:

_____________________________________________________

_____________________________________________________

_____________________________________________________

_____________________________________________________

Contact Telephone Numbers:

Number1: Contact Name_____________________

Number2: Contact Name_____________________

Number3: Contact Name_____________________

Clinic attended by client: _______________________________________

Named Research Nurse responsible for Client: ______________________

# 4. Record of Home Visits

| **Visit Name*** | **Date of Visit** | **Type of visit (Routine/ Rescheduled/ Unscheduled)** | **Visit Outcome**  **(Contact/No contact/ Refused)**** | **VLW Signature** | **Client Signature** |
| --- | --- | --- | --- | --- | --- |
|  |  |  |  |  |  |
|  |  |  |  |  |  |
|  |  |  |  |  |  |
|  |  |  |  |  |  |
|  |  |  |  |  |  |
|  |  |  |  |  |  |
|  |  |  |  |  |  |
|  |  |  |  |  |  |
|  |  |  |  |  |  |
|  |  |  |  |  |  |
|  |  |  |  |  |  |
|  |  |  |  |  |  |
|  |  |  |  |  |  |
|  |  |  |  |  |  |
|  |  |  |  |  |  |
|  |  |  |  |  |  |

**Refer to table 1.5 and 1.6 for the “Visit Name”*

***If you do a visit but the client refuses, DO record this on the chart*

# 5. Record of Individual Visits

# INITIAL VISIT

Date of visit: //

Individuals present in the Discussion: ……………….……………………………………………………….

Length of visit: ................................................................................................................

**Objectives of visit**

- Confirm the address that client provided at recruitment
- Make initial contact with the client
- Describe the home visit intervention

**Activities to complete**

□ Confirm the client’s address and telephone number is correct

□ Introduce yourself to the client

□ Explain the home visits to client: purpose, structure, logistics

**Explanatory notes**

This is very important visit as the client will make their first impression of you and this can be an important determinant of the development of a relationship.

**Pre-visit information from Clinic Staff**

__________________________________________________________

__________________________________________________________

__________________________________________________________

__________________________________________________________

**Main points of visit / Issues Discussed**

__________________________________________________________

__________________________________________________________

__________________________________________________________

__________________________________________________________

__________________________________________________________

__________________________________________________________

__________________________________________________________

**Specific problems or concerns Identified by Client**

__________________________________________________________

__________________________________________________________

__________________________________________________________

__________________________________________________________

**Action points agreed with the Client**

__________________________________________________________

__________________________________________________________

__________________________________________________________

__________________________________________________________

**Issues to follow-up**

__________________________________________________________

__________________________________________________________

__________________________________________________________

__________________________________________________________

**Comments/issues about the visit** __________________________________________________________

__________________________________________________________

__________________________________________________________

# INTRODUCTORY VISIT

Cxxq22222222222222222222222222Date of visit: //

Individuals present in the Discussion: ……………….……………………………………………………….

Length of visit: ................................................................................................................

**Objectives of visit**

- Give information on HIV
- Answering questions arising from the clinic appointment
- Psychosocial assessment & family mapping
- Assessment of disclosure
- Assess family testing needs

**Activities to complete**

□ Give information about HIV infection, its treatment, management of HIV within the home, and any useful resources relevant to the age-group of the client

□ Answer questions from the clinical appointment regarding HIV, its treatment, monitoring,

□ Family mapping: Identify primary and secondary caregivers, their relationship to the child, and whether any household members are HIV+ve /already on ART

□ Help client to identify the strengths and resources available to them

□ Assess what has been told to the child about their HIV status

□ Check who else in the family/community/school has been told the child’s diagnosis

□ Check who has been tested in the family and which family members need to be tested (parents, siblings, caregiver) & give information about benefits of HIV testing

**Explanatory notes**

Use the “Helping Caregivers to Identify their strengths and skills and support networks” (Page 65) for family mapping and helping clients identify their strengths and resources. These can be referred to in subsequent sessions when needed.

**Pre-visit information from Clinic Staff**

__________________________________________________________

__________________________________________________________

__________________________________________________________

__________________________________________________________

**Main points of visit / Issues Discussed**

__________________________________________________________

__________________________________________________________

__________________________________________________________

__________________________________________________________

__________________________________________________________

__________________________________________________________

__________________________________________________________

**Specific problems or concerns Identified by Client**

__________________________________________________________

__________________________________________________________

__________________________________________________________

__________________________________________________________

**Action points agreed with the Client**

__________________________________________________________

__________________________________________________________

__________________________________________________________

__________________________________________________________

**Issues to follow-up**

__________________________________________________________

__________________________________________________________

__________________________________________________________

__________________________________________________________

**Comments/issues about the visit** __________________________________________________________

__________________________________________________________

__________________________________________________________

# PLANNING FOR SUCCESSFUL TREATMENT

Date of visit: //

Individuals present in the Discussion: ……………….……………………………………………………….

Length of visit: ................................................................................................................

**Objectives of visit**

- Discussion of treatment experience to date
- Development of a Personal Treatment Plan
- Linkage to locally available support services
- Discussion of management of drug stock-outs
- Follow-up on disclosure to child/others
- Follow-up on family testing

**Activities to complete**

□ Discuss treatment experience so far

□ Develop a Personal Treatment Plan with the caregiver (and/or child, depending on age and development), drawing on strengths and resources previously identified

*(If Introductory visit happened a long time ago, you may need to re-identify strengths and resources available to child, caregiver and household)*

□ Answer general questions as well as specific ones emerging from clinical appointments

□ Discuss how to manage drug stock-outs or additional treatment charges

□ Assess need/eligibility for referrals to locally available services, support groups, etc. and provide information on organisations active in the area and facilitate referral (or follow-up on referrals previously made/taken up)

□ Follow-up on any changes in disclosure to child/others

□ Follow-up on testing uptake by other family members and record if anyone within the family has tested

**Explanatory notes**

- Use the guide on “Developing a Personal Treatment Plan” (Page 70-73). Two copies of the Personal Treatment plan are provided. Use the example copy (Page 72) to explain Treatment planning to the client and this copy should stay in the manual. Use the other copy (page 73) to draw up a plan with the client and pull out from manual and give this to the client to keep, You and the client should refer to this at every home visit.
- Discuss specific tools for adherence and which strategies the client think may be useful. Encourage them to come up with their own ideas.
- You may need to go over requirements of regimen and how drugs work as clients can become overloaded with information and often forget information given at clinic

**Pre-visit information from Clinic Staff**

__________________________________________________________

__________________________________________________________

__________________________________________________________

__________________________________________________________

**Main points of visit / Issues Discussed**

__________________________________________________________

__________________________________________________________

__________________________________________________________

__________________________________________________________

__________________________________________________________

__________________________________________________________

__________________________________________________________

**Specific problems or concerns Identified by Client**

__________________________________________________________

__________________________________________________________

__________________________________________________________

__________________________________________________________

**Action points agreed with the Client**

__________________________________________________________

__________________________________________________________

__________________________________________________________

__________________________________________________________

**Issues to follow-up**

__________________________________________________________

__________________________________________________________

__________________________________________________________

__________________________________________________________

**Comments/issues about the visit** __________________________________________________________

__________________________________________________________

__________________________________________________________

# REVIEW OF SIDE EFFECTS

Date of visit: //

Individuals present in the Discussion: ……………….……………………………………………………….

Length of visit: ................................................................................................................

**Objectives of visit**

- Review of Treatment plan
- Discussion on side effects and management strategies
- Follow-up on any changes in disclosure to child/others
- Follow-up on testing uptake by other household members

**Activities to complete**

□ Review Personal Treatment Plan, and discuss how well it works and what changes are required

□ Have a discussion around side effects: any side-effects so far and how to manage them

□ Provide information on managing side effects, including age-appropriate factsheets

□ Answer general questions as well as specific ones emerging from clinical appointments

□ Follow-up on referrals made at previous visit, and whether these have been taken up, need assistance, or new referrals are required

□ Follow-up on any changes in disclosure to child/others

□ Follow-up on testing uptake by other family members and record if anyone within the family has tested

**Explanatory notes**

Record specific side-effects experienced by the client

Whenever you discuss the Treatment plan, ask the client to bring out the Personal treatment Plan you drew up on the earlier visit. The emphasis is to really probe in detail around daily routines, ways to remind caregivers about the time to take the drugs, and how realistically to get others to help and avoid obstacles like having other people in the house to whom the family doesn't want to disclose - there have to be ways around this, and with a bit of forward planning and building confidence, most barriers can be identified and strategies for avoiding or making them less problematic and put into place. Key idea is that these strategies should be "realistic" for the family.

**Pre-visit information from Clinic Staff**

__________________________________________________________

__________________________________________________________

__________________________________________________________

__________________________________________________________

**Main points of visit / Issues Discussed**

__________________________________________________________

__________________________________________________________

__________________________________________________________

__________________________________________________________

__________________________________________________________

__________________________________________________________

__________________________________________________________

**Specific problems or concerns Identified by Client**

__________________________________________________________

__________________________________________________________

__________________________________________________________

__________________________________________________________

**Action points agreed with the Client**

__________________________________________________________

__________________________________________________________

__________________________________________________________

__________________________________________________________

**Issues to follow-up**

__________________________________________________________

__________________________________________________________

__________________________________________________________

__________________________________________________________

**Comments/issues about the visit** __________________________________________________________

__________________________________________________________

__________________________________________________________

# DISCLOSURE

Date of visit: //

Individuals present in the Discussion: ……………….……………………………………………………….

Length of visit: ................................................................................................................

**Objectives of visit**

- Review of Personal Treatment Plan
- Facilitated discussion around disclosure to child
- Follow-up on referrals
- Follow-up on testing uptake by other household members

**Activities to complete**

□ Review Personal Treatment Plan, and discuss how well it works and what changes are required

□ Discuss disclosure to the child: give suggestions (and written information) on how to disclose to children at different ages; offer to assist with disclosure on next visits.

□ Answer general questions as well as specific ones emerging from clinical appointments

□ Follow-up on referrals made at previous visit, and whether these have been taken up, need assistance, or new referrals are required

□ Follow-up on testing uptake by other family members and record if anyone within the family has tested

**Explanatory notes**

- Read the “Tips in Facilitating Disclosure” (Page 64)

Explore advantages and disadvantages of disclosure and caregiver concerns around disclosure.

**Pre-visit information from Clinic Staff**

__________________________________________________________

__________________________________________________________

__________________________________________________________

__________________________________________________________

**Main points of visit / Issues Discussed**

__________________________________________________________

__________________________________________________________

__________________________________________________________

__________________________________________________________

__________________________________________________________

__________________________________________________________

__________________________________________________________

**Specific problems or concerns Identified by Client**

__________________________________________________________

__________________________________________________________

__________________________________________________________

__________________________________________________________

**Action points agreed with the Client**

__________________________________________________________

__________________________________________________________

__________________________________________________________

__________________________________________________________

**Issues to follow-up**

__________________________________________________________

__________________________________________________________

__________________________________________________________

__________________________________________________________

**Comments/issues about the visit** __________________________________________________________

__________________________________________________________

__________________________________________________________

(Page intentionally left blank)

# MAINTENANCE VISITS

This visit takes place after a longer time period than the previous home visits. Thus, there may be a “backlog” of issues to address, and it is important to really probe to find out how the past 3 months have gone and whether there have been any concerns relating to adherence, managing side effects, relationships in the family and community, etc. So the first priority is to really talk through how the past 3 months have gone, and if there are any practical ways to support the client and household.

However, at this point, the client is going to undergo a transition to 3-monthly monitoring clinical appoints and parallel home visits. They need to be prepared for this new schedule, and also it will be important to explain that this marks a new phase of taking increasing responsibility for lifelong treatment. The ZENITH programme will start to “phase out” and "hand over" to the family (and the child).

In this first visit, the concept of “maintenance” should be explained, which refers to adopting routines that will ensure adherence to medication and positive living for the rest of the child’s life. You can tell the client that for the remaining visits until the end of follow-up, you will be providing some tips on going from being "new" patients to lifelong adherents to ART. There will be many challenges ahead, especially as the child goes through adolescence, and you will be discussing these over the next few months.

It might be useful to emphasise positive experiences so far, and how the family has been able to use their strengths to commit to a treatment plan, and take advantage of other available services (give examples). This this has left them in a strong position so they can face the future on their own.

# MAINTENANCE (on ART)

Date of visit: //

Individuals present in the Discussion: ……………….……………………………………………………….

Length of visit: ................................................................................................................

**Objectives of visit**

- Review of Personal Treatment Plan
- Check on disclosure to the child
- Discussion around long-term maintenance of treatment
- Follow-up on referrals
- Follow-up on testing uptake by other household members

**Activities to complete**

□ Review Personal Treatment Plan, and discuss how well it works and what changes are required

□ Check on disclosure to child: check on the reactions of the child and any emerging issues if child aware of HIV status; offer to help with disclosure if child not yet aware

□ Have a discussion about long-term maintenance of treatment, including ongoing commitment and reminder of identified strengths & resources that will support the treatment plan

□ Answer general questions as well as specific ones emerging from clinical appointments

□ Follow-up on referrals made at previous visit, and whether these have been taken up, need assistance, or new referrals are required

□ Follow-up on testing uptake by other family members and record if anyone within the family has tested

**Explanatory notes**

Some examples of discussion points include problems with adherence including stigma, privacy, interruption of schooling, side-effects and emotional challenges such as isolation and depression.

**Pre-visit information from Clinic Staff**

__________________________________________________________

__________________________________________________________

__________________________________________________________

__________________________________________________________

**Main points of visit / Issues Discussed**

__________________________________________________________

__________________________________________________________

__________________________________________________________

__________________________________________________________

__________________________________________________________

__________________________________________________________

__________________________________________________________

**Specific problems or concerns Identified by Client**

__________________________________________________________

__________________________________________________________

__________________________________________________________

__________________________________________________________

**Action points agreed with the Client**

__________________________________________________________

__________________________________________________________

__________________________________________________________

__________________________________________________________

**Issues to follow-up**

__________________________________________________________

__________________________________________________________

__________________________________________________________

__________________________________________________________

**Comments/issues about the visit** __________________________________________________________

__________________________________________________________

__________________________________________________________

# DISCLOSURE (not on ART)

Date of visit: //

Individuals present in the Discussion: ……………….……………………………………………………….

Length of visit: ................................................................................................................

**Objectives of visit**

- Discussion about monitoring
- Discussion about positive living
- Facilitated discussion around disclosure to child
- Linkage to locally available support services
- Follow-up on testing uptake by other household members

**Activities to complete**

□ Review Personal Treatment Plan, and discuss how well it works and what changes are required

□ Discuss disclosure to the child: give suggestions (and written information) on how to disclose to children at different ages; offer to assist with disclosure on next visits.

□ Answer general questions as well as specific ones emerging from clinical appointments

□ Follow-up on referrals made at previous visit, and whether these have been taken up, need assistance, or new referrals are required

□ Follow-up on testing uptake by other family members and record if anyone within the family has tested

**Explanatory notes**

- Read the “Tips in Facilitating Disclosure” (Page 64)

Explore advantages and disadvantages of disclosure and caregiver concerns around disclosure.

**Pre-visit information from Clinic Staff**

__________________________________________________________

__________________________________________________________

__________________________________________________________

__________________________________________________________

**Main points of visit / Issues Discussed**

__________________________________________________________

__________________________________________________________

__________________________________________________________

__________________________________________________________

__________________________________________________________

__________________________________________________________

__________________________________________________________

**Specific problems or concerns Identified by Client**

__________________________________________________________

__________________________________________________________

__________________________________________________________

__________________________________________________________

**Action points agreed with the Client**

__________________________________________________________

__________________________________________________________

__________________________________________________________

__________________________________________________________

**Issues to follow-up**

__________________________________________________________

__________________________________________________________

__________________________________________________________

__________________________________________________________

**Comments/issues about the visit** __________________________________________________________

__________________________________________________________

__________________________________________________________

# ONGOING SUPPORT (on ART)

Date of visit: //

Individuals present in the Discussion: ……………….……………………………………………………….

Length of visit: ................................................................................................................

**Objective of visit**

- Review Personal treatment Plan
- Follow-up on issues emerging from clinical monitoring appointments
- Review: referrals, disclosure, support/testing of household members
- Address client concerns

**Activities to complete**

□ Review Personal Treatment Plan and experience with treatment

□ Discuss and follow-up on issues emerging from clinical monitoring appointments

□ Review need for referrals, assistance with disclosure, and/or support for testing of other household members

□ Answer questions, facilitating identification of solutions to emerging challenges, and provide relevant information

**Explanatory notes**

Identify specific problems with adherence and side-effects through detailed discussion. Facilitate creation of solutions.

**Pre-visit information from Clinic Staff**

__________________________________________________________

__________________________________________________________

__________________________________________________________

__________________________________________________________

**Main points of visit / Issues Discussed**

__________________________________________________________

__________________________________________________________

__________________________________________________________

__________________________________________________________

__________________________________________________________

__________________________________________________________

__________________________________________________________

**Specific problems or concerns Identified by Client**

__________________________________________________________

__________________________________________________________

__________________________________________________________

__________________________________________________________

**Action points agreed with the Client**

__________________________________________________________

__________________________________________________________

__________________________________________________________

__________________________________________________________

**Issues to follow-up**

__________________________________________________________

__________________________________________________________

__________________________________________________________

__________________________________________________________

**Comments/issues about the visit** __________________________________________________________

__________________________________________________________

__________________________________________________________

# ONGOING SUPPORT (on ART)

Date of visit: //

Individuals present in the Discussion: ……………….……………………………………………………….

Length of visit: ................................................................................................................

**Objective of visit**

- Review Personal treatment Plan
- Follow-up on issues emerging from clinical monitoring appointments
- Review: referrals, disclosure, support/testing of household members
- Address client concerns

**Activities to complete**

□ Review Personal Treatment Plan and experience with treatment

□ Discuss and follow-up on issues emerging from clinical monitoring appointments

□ Review need for referrals, assistance with disclosure, and/or support for testing of other household members

□ Answer questions, facilitating identification of solutions to emerging challenges, and provide relevant information

**Explanatory notes**

Identify specific problems with adherence and side-effects through detailed discussion. Facilitate creation of solutions.

**Pre-visit information from Clinic Staff**

__________________________________________________________

__________________________________________________________

__________________________________________________________

__________________________________________________________

**Main points of visit / Issues Discussed**

__________________________________________________________

__________________________________________________________

__________________________________________________________

__________________________________________________________

__________________________________________________________

__________________________________________________________

__________________________________________________________

**Specific problems or concerns Identified by Client**

__________________________________________________________

__________________________________________________________

__________________________________________________________

__________________________________________________________

**Action points agreed with the Client**

__________________________________________________________

__________________________________________________________

__________________________________________________________

__________________________________________________________

**Issues to follow-up**

__________________________________________________________

__________________________________________________________

__________________________________________________________

__________________________________________________________

**Comments/issues about the visit** __________________________________________________________

__________________________________________________________

__________________________________________________________

# ONGOING SUPPORT (on ART)

Date of visit: //

Individuals present in the Discussion: ……………….……………………………………………………….

Length of visit: ................................................................................................................

**Objective of visit**

- Review Personal treatment Plan
- Follow-up on issues emerging from clinical monitoring appointments
- Review: referrals, disclosure, support/testing of household members
- Address client concerns

**Activities to complete**

□ Review Personal Treatment Plan and experience with treatment

□ Discuss and follow-up on issues emerging from clinical monitoring appointments

□ Review need for referrals, assistance with disclosure, and/or support for testing of other household members

□ Answer questions, facilitating identification of solutions to emerging challenges, and provide relevant information

**Explanatory notes**

Identify specific problems with adherence and side-effects through detailed discussion. Facilitate creation of solutions.

**Pre-visit information from Clinic Staff**

__________________________________________________________

__________________________________________________________

__________________________________________________________

__________________________________________________________

**Main points of visit / Issues Discussed**

__________________________________________________________

__________________________________________________________

__________________________________________________________

__________________________________________________________

__________________________________________________________

__________________________________________________________

__________________________________________________________

**Specific problems or concerns Identified by Client**

__________________________________________________________

__________________________________________________________

__________________________________________________________

__________________________________________________________

**Action points agreed with the Client**

__________________________________________________________

__________________________________________________________

__________________________________________________________

__________________________________________________________

**Issues to follow-up**

__________________________________________________________

__________________________________________________________

__________________________________________________________

__________________________________________________________

**Comments/issues about the visit** __________________________________________________________

__________________________________________________________

__________________________________________________________

# ONGOING SUPPORT (on ART)

Date of visit: //

Individuals present in the Discussion: ……………….……………………………………………………….

Length of visit: ................................................................................................................

**Objective of visit**

- Review Personal treatment Plan
- Follow-up on issues emerging from clinical monitoring appointments
- Review: referrals, disclosure, support/testing of household members
- Address client concerns

**Activities to complete**

□ Review Personal Treatment Plan and experience with treatment

□ Discuss and follow-up on issues emerging from clinical monitoring appointments

□ Review need for referrals, assistance with disclosure, and/or support for testing of other household members

□ Answer questions, facilitating identification of solutions to emerging challenges, and provide relevant information

**Explanatory notes**

Identify specific problems with adherence and side-effects through detailed discussion. Facilitate creation of solutions.

**Pre-visit information from Clinic Staff**

__________________________________________________________

__________________________________________________________

__________________________________________________________

__________________________________________________________

**Main points of visit / Issues Discussed**

__________________________________________________________

__________________________________________________________

__________________________________________________________

__________________________________________________________

__________________________________________________________

__________________________________________________________

__________________________________________________________

**Specific problems or concerns Identified by Client**

__________________________________________________________

__________________________________________________________

__________________________________________________________

__________________________________________________________

**Action points agreed with the Client**

__________________________________________________________

__________________________________________________________

__________________________________________________________

__________________________________________________________

**Issues to follow-up**

__________________________________________________________

__________________________________________________________

__________________________________________________________

__________________________________________________________

**Comments/issues about the visit** __________________________________________________________

__________________________________________________________

__________________________________________________________

# ONGOING SUPPORT (on ART)

Date of visit: //

Individuals present in the Discussion: ……………….……………………………………………………….

Length of visit: ................................................................................................................

**Objective of visit**

- Review Personal treatment Plan
- Follow-up on issues emerging from clinical monitoring appointments
- Review: referrals, disclosure, support/testing of household members
- Address client concerns

**Activities to complete**

□ Review Personal Treatment Plan and experience with treatment

□ Discuss and follow-up on issues emerging from clinical monitoring appointments

□ Review need for referrals, assistance with disclosure, and/or support for testing of other household members

□ Answer questions, facilitating identification of solutions to emerging challenges, and provide relevant information

**Explanatory notes**

Identify specific problems with adherence and side-effects through detailed discussion. Facilitate creation of solutions.

**Pre-visit information from Clinic Staff**

__________________________________________________________

__________________________________________________________

__________________________________________________________

__________________________________________________________

**Main points of visit / Issues Discussed**

__________________________________________________________

__________________________________________________________

__________________________________________________________

__________________________________________________________

__________________________________________________________

__________________________________________________________

__________________________________________________________

**Specific problems or concerns Identified by Client**

__________________________________________________________

__________________________________________________________

__________________________________________________________

__________________________________________________________

**Action points agreed with the Client**

__________________________________________________________

__________________________________________________________

__________________________________________________________

__________________________________________________________

**Issues to follow-up**

__________________________________________________________

__________________________________________________________

__________________________________________________________

__________________________________________________________

**Comments/issues about the visit** __________________________________________________________

__________________________________________________________

__________________________________________________________

# ONGOING SUPPORT (on ART)

Date of visit: //

Individuals present in the Discussion: ……………….……………………………………………………….

Length of visit: ................................................................................................................

**Objective of visit**

- Review Personal treatment Plan
- Follow-up on issues emerging from clinical monitoring appointments
- Review: referrals, disclosure, support/testing of household members
- Address client concerns

**Activities to complete**

□ Review Personal Treatment Plan and experience with treatment

□ Discuss and follow-up on issues emerging from clinical monitoring appointments

□ Review need for referrals, assistance with disclosure, and/or support for testing of other household members

□ Answer questions, facilitating identification of solutions to emerging challenges, and provide relevant information

**Explanatory notes**

Identify specific problems with adherence and side-effects through detailed discussion. Facilitate creation of solutions.

**Pre-visit information from Clinic Staff**

__________________________________________________________

__________________________________________________________

__________________________________________________________

__________________________________________________________

**Main points of visit / Issues Discussed**

__________________________________________________________

__________________________________________________________

__________________________________________________________

__________________________________________________________

__________________________________________________________

__________________________________________________________

__________________________________________________________

**Specific problems or concerns Identified by Client**

__________________________________________________________

__________________________________________________________

__________________________________________________________

__________________________________________________________

**Action points agreed with the Client**

__________________________________________________________

__________________________________________________________

__________________________________________________________

__________________________________________________________

**Issues to follow-up**

__________________________________________________________

__________________________________________________________

__________________________________________________________

__________________________________________________________

**Comments/issues about the visit** __________________________________________________________

__________________________________________________________

__________________________________________________________

# ONGOING SUPPORT (not on ART)

Date of visit: //

Individuals present in the Discussion: ……………….……………………………………………………….

Length of visit: ................................................................................................................

**Objective of visit**

- Follow-up on issues emerging from clinical monitoring appointments
- Review: referrals, disclosure, support/testing of household members
- Encouraging maintenance of regular clinic attendance
- Address positive living

**Activities to complete**

□ Follow up on any issues that arise from clinical appointments at PHC

□ Answer general questions as well as specific ones emerging from clinical appointments

□ Emphasize positive living and how to remain without treatment

□ Reinforce the importance of maintaining monitoring appointments to check for eligibility and to manage other health issues

□ Review need for referrals, assistance with disclosure, and/or support for testing of other household members

**Explanatory notes**

Refer to “Tips for Positive Living” (Page 69), to help advise the client when you are discussing positive living with them.

.

**Pre-visit information from Clinic Staff**

__________________________________________________________

__________________________________________________________

__________________________________________________________

__________________________________________________________

**Main points of visit / Issues Discussed**

__________________________________________________________

__________________________________________________________

__________________________________________________________

__________________________________________________________

__________________________________________________________

__________________________________________________________

__________________________________________________________

**Specific problems or concerns Identified by Client**

__________________________________________________________

__________________________________________________________

__________________________________________________________

__________________________________________________________

**Action points agreed with the Client**

__________________________________________________________

__________________________________________________________

__________________________________________________________

__________________________________________________________

**Issues to follow-up**

__________________________________________________________

__________________________________________________________

__________________________________________________________

__________________________________________________________

**Comments/issues about the visit** __________________________________________________________

__________________________________________________________

__________________________________________________________

# ONGOING SUPPORT (not on ART)

Date of visit: //

Individuals present in the Discussion: ……………….……………………………………………………….

Length of visit: ................................................................................................................

**Objective of visit**

- Follow-up on issues emerging from clinical monitoring appointments
- Review: referrals, disclosure, support/testing of household members
- Encouraging maintenance of regular clinic attendance
- Address positive living

**Activities to complete**

□ Follow up on any issues that arise from clinical appointments at PHC

□ Answer general questions as well as specific ones emerging from clinical appointments

□ Emphasize positive living and how to remain without treatment

□ Reinforce the importance of maintaining monitoring appointments to check for eligibility and to manage other health issues

□ Review need for referrals, assistance with disclosure, and/or support for testing of other household members

**Explanatory notes**

**Pre-visit information from Clinic Staff**

__________________________________________________________

__________________________________________________________

__________________________________________________________

__________________________________________________________

**Main points of visit / Issues Discussed**

__________________________________________________________

__________________________________________________________

__________________________________________________________

__________________________________________________________

__________________________________________________________

__________________________________________________________

__________________________________________________________

**Specific problems or concerns Identified by Client**

__________________________________________________________

__________________________________________________________

__________________________________________________________

__________________________________________________________

**Action points agreed with the Client**

__________________________________________________________

__________________________________________________________

__________________________________________________________

__________________________________________________________

**Issues to follow-up**

__________________________________________________________

__________________________________________________________

__________________________________________________________

__________________________________________________________

**Comments/issues about the visit** __________________________________________________________

__________________________________________________________

__________________________________________________________

# ONGOING SUPPORT (not on ART)

Date of visit: //

Individuals present in the Discussion: ……………….……………………………………………………….

Length of visit: ................................................................................................................

**Objective of visit**

- Follow-up on issues emerging from clinical monitoring appointments
- Review: referrals, disclosure, support/testing of household members
- Encouraging maintenance of regular clinic attendance
- Address positive living

**Activities to complete**

□ Follow up on any issues that arise from clinical appointments at PHC

□ Answer general questions as well as specific ones emerging from clinical appointments

□ Emphasize positive living and how to remain without treatment

□ Reinforce the importance of maintaining monitoring appointments to check for eligibility and to manage other health issues

□ Review need for referrals, assistance with disclosure, and/or support for testing of other household members

**Explanatory notes**

**Pre-visit information from Clinic Staff**

__________________________________________________________

__________________________________________________________

__________________________________________________________

__________________________________________________________

**Main points of visit / Issues Discussed**

__________________________________________________________

__________________________________________________________

__________________________________________________________

__________________________________________________________

__________________________________________________________

__________________________________________________________

__________________________________________________________

**Specific problems or concerns Identified by Client**

__________________________________________________________

__________________________________________________________

__________________________________________________________

__________________________________________________________

**Action points agreed with the Client**

__________________________________________________________

__________________________________________________________

__________________________________________________________

__________________________________________________________

**Issues to follow-up**

__________________________________________________________

__________________________________________________________

__________________________________________________________

__________________________________________________________

**Comments/issues about the visit** __________________________________________________________

__________________________________________________________

__________________________________________________________

# ONGOING SUPPORT (not on ART)

Date of visit: //

Individuals present in the Discussion: ……………….……………………………………………………….

Length of visit: ................................................................................................................

**Objective of visit**

- Follow-up on issues emerging from clinical monitoring appointments
- Review: referrals, disclosure, support/testing of household members
- Encouraging maintenance of regular clinic attendance
- Address positive living

**Activities to complete**

□ Follow up on any issues that arise from clinical appointments at PHC

□ Answer general questions as well as specific ones emerging from clinical appointments

□ Emphasize positive living and how to remain without treatment

□ Reinforce the importance of maintaining monitoring appointments to check for eligibility and to manage other health issues

□ Review need for referrals, assistance with disclosure, and/or support for testing of other household members

**Explanatory notes**

**Pre-visit information from Clinic Staff**

__________________________________________________________

__________________________________________________________

__________________________________________________________

__________________________________________________________

**Main points of visit / Issues Discussed**

__________________________________________________________

__________________________________________________________

__________________________________________________________

__________________________________________________________

__________________________________________________________

__________________________________________________________

__________________________________________________________

**Specific problems or concerns Identified by Client**

__________________________________________________________

__________________________________________________________

__________________________________________________________

__________________________________________________________

**Action points agreed with the Client**

__________________________________________________________

__________________________________________________________

__________________________________________________________

__________________________________________________________

**Issues to follow-up**

__________________________________________________________

__________________________________________________________

__________________________________________________________

__________________________________________________________

**Comments/issues about the visit** __________________________________________________________

__________________________________________________________

__________________________________________________________

# ONGOING SUPPORT (not on ART)

Date of visit: //

Individuals present in the Discussion: ……………….……………………………………………………….

Length of visit: ................................................................................................................

**Objective of visit**

- Follow-up on issues emerging from clinical monitoring appointments
- Review: referrals, disclosure, support/testing of household members
- Encouraging maintenance of regular clinic attendance
- Address positive living

**Activities to complete**

□ Follow up on any issues that arise from clinical appointments at PHC

□ Answer general questions as well as specific ones emerging from clinical appointments

□ Emphasize positive living and how to remain without treatment

□ Reinforce the importance of maintaining monitoring appointments to check for eligibility and to manage other health issues

□ Review need for referrals, assistance with disclosure, and/or support for testing of other household members

**Explanatory notes**

**Pre-visit information from Clinic Staff**

__________________________________________________________

__________________________________________________________

__________________________________________________________

__________________________________________________________

**Main points of visit / Issues Discussed**

__________________________________________________________

__________________________________________________________

__________________________________________________________

__________________________________________________________

__________________________________________________________

__________________________________________________________

__________________________________________________________

**Specific problems or concerns Identified by Client**

__________________________________________________________

__________________________________________________________

__________________________________________________________

__________________________________________________________

**Action points agreed with the Client**

__________________________________________________________

__________________________________________________________

__________________________________________________________

__________________________________________________________

**Issues to follow-up**

__________________________________________________________

__________________________________________________________

__________________________________________________________

__________________________________________________________

**Comments/issues about the visit** __________________________________________________________

__________________________________________________________

__________________________________________________________

# ONGOING SUPPORT (not on ART)

Date of visit: //

Individuals present in the Discussion: ……………….……………………………………………………….

Length of visit: ................................................................................................................

**Objective of visit**

- Follow-up on issues emerging from clinical monitoring appointments
- Review: referrals, disclosure, support/testing of household members
- Encouraging maintenance of regular clinic attendance
- Address positive living

**Activities to complete**

□ Follow up on any issues that arise from clinical appointments at PHC

□ Answer general questions as well as specific ones emerging from clinical appointments

□ Emphasize positive living and how to remain without treatment

□ Reinforce the importance of maintaining monitoring appointments to check for eligibility and to manage other health issues

□ Review need for referrals, assistance with disclosure, and/or support for testing of other household members

**Explanatory notes**

**Pre-visit information from Clinic Staff**

__________________________________________________________

__________________________________________________________

__________________________________________________________

__________________________________________________________

**Main points of visit / Issues Discussed**

__________________________________________________________

__________________________________________________________

__________________________________________________________

__________________________________________________________

__________________________________________________________

__________________________________________________________

__________________________________________________________

**Specific problems or concerns Identified by Client**

__________________________________________________________

__________________________________________________________

__________________________________________________________

__________________________________________________________

**Action points agreed with the Client**

__________________________________________________________

__________________________________________________________

__________________________________________________________

__________________________________________________________

**Issues to follow-up**

__________________________________________________________

__________________________________________________________

__________________________________________________________

__________________________________________________________

**Comments/issues about the visit** __________________________________________________________

__________________________________________________________

__________________________________________________________

# ONGOING SUPPORT (not on ART)

Date of visit: //

Individuals present in the Discussion: ……………….……………………………………………………….

Length of visit: ................................................................................................................

**Objective of visit**

- Follow-up on issues emerging from clinical monitoring appointments
- Review: referrals, disclosure, support/testing of household members
- Encouraging maintenance of regular clinic attendance
- Address positive living

**Activities to complete**

□ Follow up on any issues that arise from clinical appointments at PHC

□ Answer general questions as well as specific ones emerging from clinical appointments

□ Emphasize positive living and how to remain without treatment

□ Reinforce the importance of maintaining monitoring appointments to check for eligibility and to manage other health issues

□ Review need for referrals, assistance with disclosure, and/or support for testing of other household members

**Explanatory notes**

**Pre-visit information from Clinic Staff**

__________________________________________________________

__________________________________________________________

__________________________________________________________

__________________________________________________________

**Main points of visit / Issues Discussed**

__________________________________________________________

__________________________________________________________

__________________________________________________________

__________________________________________________________

__________________________________________________________

__________________________________________________________

__________________________________________________________

**Specific problems or concerns Identified by Client**

__________________________________________________________

__________________________________________________________

__________________________________________________________

__________________________________________________________

**Action points agreed with the Client**

__________________________________________________________

__________________________________________________________

__________________________________________________________

__________________________________________________________

**Issues to follow-up**

__________________________________________________________

__________________________________________________________

__________________________________________________________

__________________________________________________________

**Comments/issues about the visit** __________________________________________________________

__________________________________________________________

__________________________________________________________

# ONGOING SUPPORT (not on ART)

Date of visit: //

Individuals present in the Discussion: ……………….……………………………………………………….

Length of visit: ................................................................................................................

**Objective of visit**

- Follow-up on issues emerging from clinical monitoring appointments
- Review: referrals, disclosure, support/testing of household members
- Encouraging maintenance of regular clinic attendance
- Address positive living

**Activities to complete**

□ Follow up on any issues that arise from clinical appointments at PHC

□ Answer general questions as well as specific ones emerging from clinical appointments

□ Emphasize positive living and how to remain without treatment

□ Reinforce the importance of maintaining monitoring appointments to check for eligibility and to manage other health issues

□ Review need for referrals, assistance with disclosure, and/or support for testing of other household members

**Explanatory notes**

**Pre-visit information from Clinic Staff**

__________________________________________________________

__________________________________________________________

__________________________________________________________

__________________________________________________________

**Main points of visit / Issues Discussed**

__________________________________________________________

__________________________________________________________

__________________________________________________________

__________________________________________________________

__________________________________________________________

__________________________________________________________

__________________________________________________________

**Specific problems or concerns Identified by Client**

__________________________________________________________

__________________________________________________________

__________________________________________________________

__________________________________________________________

**Action points agreed with the Client**

__________________________________________________________

__________________________________________________________

__________________________________________________________

__________________________________________________________

**Issues to follow-up**

__________________________________________________________

__________________________________________________________

__________________________________________________________

__________________________________________________________

**Comments/issues about the visit** __________________________________________________________

__________________________________________________________

__________________________________________________________

# UNSCHEDULED VISIT

Date of visit: //

Individuals present in the Discussion: ……………….……………………………………………………….

**Why did you make this visit?**

__________________________________________________________

__________________________________________________________

__________________________________________________________

__________________________________________________________

**Main points of visit / Issues Discussed**

__________________________________________________________

__________________________________________________________

__________________________________________________________

__________________________________________________________

__________________________________________________________

__________________________________________________________

__________________________________________________________

**Action points agreed with the Client**

__________________________________________________________

__________________________________________________________

__________________________________________________________

__________________________________________________________

**Issues to follow-up**

__________________________________________________________

__________________________________________________________

__________________________________________________________

__________________________________________________________

**Comments/issues about the visit** __________________________________________________________

__________________________________________________________

__________________________________________________________

__________________________________________________________

# UNSCHEDULED VISIT

Date of visit: //

Individuals present in the Discussion: ……………….……………………………………………………….

**Why did you make this visit?**

__________________________________________________________

__________________________________________________________

__________________________________________________________

__________________________________________________________

**Main points of visit / Issues Discussed**

__________________________________________________________

__________________________________________________________

__________________________________________________________

__________________________________________________________

__________________________________________________________

__________________________________________________________

__________________________________________________________

**Action points agreed with the Client**

__________________________________________________________

__________________________________________________________

__________________________________________________________

__________________________________________________________

**Issues to follow-up**

__________________________________________________________

__________________________________________________________

__________________________________________________________

__________________________________________________________

**Comments/issues about the visit** __________________________________________________________

__________________________________________________________

__________________________________________________________

__________________________________________________________

# 6. Additional Information

## 6.1 COMMUNITY BASED ORGANISATIONS (CBOs) AND SUPORT GROUPS IN STUDY SUBURBS

| **Suburb** | **Name of CBO** | **Contact Person** | **Contact Number** | **Comments** |
| --- | --- | --- | --- | --- |
| Kuwadzana | Kuwirirana Support Group | Mr Madziva | 0773 479 479 | Children /adolescents meet Saturday from 1000hrs.  Provide meals for children  Africaid meets the children 1^st^ Saturday of month. |
| Kuwadzana Extension | Tamuka Foundation | Mrs Kwati | 0772 723 553 | Adolescents meet every Saturday from 0900hrs for different activities. |
| Kuwadzana | ADRA | Clinic Staff | Kuwadzana Clinic | Use of Body Mass index to determine those in need of food supplement. Referred to Dzivaresekwa clinic where ADRA is based |
| Dzivaresekwa | ADRA | Tafadzwa Chigwere | 0773 195 031 | Give food vouchers per BMI  Bus fare for the needy.  Same people give food packs to Kuwadzana deserving people |
| Dzivaresekwa | Adolescent Support Group | Adelaide | 0774 067 648 | Adolescent Support Group Chair |
| Mufakose | New Dawn Of Hope | Tariro Kutadza | 0773 103 624 | Provide psychosocial support and home based care. Provides food at times |
| Budiriro | Habitat for all | Spiwe Chabikwa  Mrs Mazikana  (social worker Budiriro Community who refers to SOS) | 0775 478 468  0733 681 094 | No active CBO except group of PLWHIV.  Social worker can be contacted for further referral to CBOs as she has all information of CBOs giving help information at any given time  SOS helps those in need of school fees but are referred by social worker. |
| Highfield | ADRA | Clinic |  | Give food using BMI |
| Highfield | Young People We Care | Brian Jack | 0733 493 681 | Adolescent support group.  Psychosocial support given |
| Glen View | Adolescent Expert | Charles Kofi |  | Psychosocial support |
| Glen View | CPS -FICA | SIC-FHS | 0772 432 202 | Money for bus fare to go for treatments |
| Glen View | ZAVEA | Sr Nyika | 0772 432 202 | Money and food following recommendations from SIC. |

## 6.2 KEY OBJECTIVES OF HOME VISITS

Although each home visit that you make to a family has specific activities and will cover some new topics, *all* the visits will require some basic tasks. These are listed below, and can be reviewed before you enter the household. These objectives should remind you to pay attention *at every visit* to how members of the household are feeling, communicating, and relating to each other. This will help you to respond to the challenges or difficulties faced by that particular family.

In each visit, you have an opportunity to build a closer relationship with the child and caregiver (and perhaps other members of the family), so that you are able to see whether they need additional assistance or extra support. The home visits have been designed to be flexible, so sometimes you may need to discuss topics that you already covered in a previous visit or check whether the caregiver understands everything you have already talked about and is able to keep up with the new issues that you bring up.

1. **ASSESSING *-* is there anything new or different in the household since your last visit?**

At each visit, you should notice the mood of the caregiver and child, and whether they seem to be facing any new problems. Even though you will have done a formal assessment of their needs before, things can change over time. Be sure to ask questions to check how things are going.

1. **PLANNING – what strategies can the caregiver use to help them ensure the child’s ongoing care?**

Over time, you might notice that the challenges faced by families may be different from before. For example, there could be changes as children become adolescents. They may start to be embarrassed about their HIV status, and more rebellious at home, including not taking treatment. You can discuss ways for the caregiver to handle new situations and encourage them to reflect on their strengths and support networks again. Reviewing the Treatment Plan will help “refresh” the caregiver’s (and child’s) planning skills.

1. **REFERRING – Could the family benefit from available services from local organisations?**

If family circumstances change, the caregiver might need referrals that were not useful for them before. You should remind caregivers of the available services and local organisations, and give them examples of how these have helped other families and why they might be useful for them now.

1. **MONITORING – Is the caregiver attending their clinical appointments and do they understand the process?**

During your regular meetings with clinic staff, you will learn whether the child has been attending his/her clinic appointments, and if there are any concerns about adherence. These can be discussed with the caregiver, and even if there do not appear to be any problems, it can be useful to check that the caregiver understands the process of clinical monitoring, and answer emerging questions. For example, they may not understand why appointments have become less frequent, or why a particular child is not yet eligible for ART. You should also check that they remember the date of their next appointment.

1. **RECOMMENDING – Is the family taking positive action on disclosure and HIV testing?**

If children know their own status and that of their caregiver, their ability to manage their own care is improved. It also helps with communication and relationships in the family if all the members are aware of their own status, and can get treatment if they need it. It is important to emphasise these messages at each meeting. Do not pressure anyone to disclose or get tested, but gently remind caregivers of how important these steps are, and help them take action when they feel ready.

## 6.3 KEY HOME VISIT SKILLS

(Adapted from Anti-Retroviral Treatment and Access to Services (ARTAS) – CDC)

- ***Make time for the client***. Try to schedule home visits at a time when you know you do not have to hurry off. Visits will take between 45 minutes and an hour and a half! You need time at each visit to build rapport, make the caregiver comfortable, find a quiet and private place to talk, and then work through the topics and activities from the manual. If you rush, the caregiver will not feel able to discuss some of the more difficult issues with you.
- ***Follow-up consistently.*** The purpose of the home visits is to encourage caregivers to ensure the child(ren) they look after go to all their clinic appointments and take their medications regularly, and on time. You are setting an example! Be on time and don’t cancel visits unless there is an emergency. Be sure to tell the caregiver when you will be back, and remind them that you will be following-up with the household for 2 years. Your clients need to feel reassured that they can rely on you!
- ***Treat each client as an individual.*** Even though all the households that are enrolled in the intervention will receive the same kinds of visits and discuss the same topics, each person you speak to will have his or her own ideas, needs, and opinions. Let each caregiver make their own choices and take actions that are right for them. You can discuss, advise, and facilitate, but you cannot take decisions for others.
- ***Be a good listener*.** Really listen to what your clients are telling you, and listen out for what they *are not telling you* – such as their fears, insecurities, or negative thoughts. Make sure your body language is friendly, and you indicate that you are interested in what the client is telling you. Don’t just focus on your agenda and allow time for their concerns to be addressed at each session.
- ***Take good notes*.** Recording what you have heard from the client and talked about is important to help you remember what to follow-up during later visits.
- ***Go at the client’s pace.*** Some people will understand HIV and treatment issues quickly, and some will need more information and greater encouragement. With some clients, you may have to repeat the same topics over and over again. Be aware of how each visit is progressing, and whether you have been able to complete all the visit objectives.
- ***Be non-judgmental*.** Clients will not always take decisions you think are the right ones, or may have acted in ways you do not approve of. It’s OK to disagree with your clients but keep this to yourself, and try to be as supportive as possible. If you feel a client is taking action that could be *harmful* to themselves of others, discuss with your supervisor immediately after the visit.
- ***Acknowledge fears and worries***. Some clients will express concerns that may seem minor or unimportant. When people face many hardships at once, they might feel depressed and overwhelmed so that *everything* seems insurmountable. Do not dismiss any worries, just listen to them and show you understand this is what the person is feeling.

But then ....

- ***FOCUS ON STRENGTHS***! The home visits are there for support and encouragement, and to help caregivers and other family members help each other and help the child living with HIV to get good care and treatment. You cannot provide assistance all the time, so your role is to identify skills, resources, and strategies that the caregiver and child can use to maximise successful treatment. Avoid talking in negative terms – when barriers and challenges come up, immediately think about how to turn them into opportunities for positive action!

## 6.4 HELPFUL TIPS ON FACILITATING DISCLOSURE

(Adapted from the WHO Guidelines on HIV disclosure to children under the age of 12)

- ***Discuss key issues in advance****.* Discuss the advantages and disadvantages and caregivers personal fears around disclosure, as well as the problem of “unhealthy disclosure”. They can then plan how/when they would like to do it. You should be aware of whether the caregiver plans to disclose to the child by him/herself, in your presence, or whether the caregiver prefers for you to talk directly to the child. Also be sure you know in advance whether the disclosure is about the *child’s status*, the *caregiver’s status*, or both. Discuss whether the caregiver feels whether partial or full disclosure will be appropriate fort he child.
- ***Schedule an appointment with family*.** Discuss in advance whom the caregiver would like to have present (it could be just the caregiver and the child, or other family members could be invited). Both caregiver and child should feel comfortable around the people present.
- ***Pay attention to the atmosphere***, and the feelings of the people present at the meeting. Try to make everyone feel comfortable, and to reduce tensions by acting confident and friendly yourself.
- If you are giving the information, ***share the diagnosis quickly*;** do not ‘‘beat around the bush”. Remaining matter-of-fact will demonstrate that the disclosure is normal process of communication with the family. If you are not the one providing the information, gently prompt the caregiver to give the information in a clear and straightforward manner.
- ***Keep medical facts to a minimum***. Describe HIV infection as a chronic illness that is manageable with treatment. You can provide further detail over the course of time.
- ***Use appropriate language*** for the developmental level and learning abilities of the child.
- ***Use any available visual aids***, pictures, or examples that will help the child understand.
- ***Accept silence during the discussion***. Children and young people often need time to process the important information being given and reflect on their feelings
- Ask family members to ***share their feelings and support each othe****r*. Ask the child to express his or her feelings and questions. Reassure the caregiver that you will be there to address some of the child's/family member's questions, and they are not on their own.
- ***Assure the child that there will be other opportunities*** to talk about the information and ask questions in future. The post-disclosure period can be very difficult for the family and child who may experience feelings of guilt/blame/anger and isolation.
- ***Explore the child’s knowledge*** about his/her health, HIV/AIDS and other chronic illnesses, and provide any additional information that seems appropriate at the time, and correct any misconceptions.

## 6.5 HELPING CAREGIVERS IDENTIFY THEIR STRENGTHS AND SKILLS AND SUPPORT NETWORKS

**Principles of the Strengths-based approach**

1. People have their *own inner resources* and the *capacity to cope* and fix their own personal challenges. They may need help, but they should be allowed to *solve their problems independently* and make their own choices that are right for them.
2. People must be *active participants* in their own change. Decisions and actions will be more effective and sustainable when people make them themselves, when they feel ready to.
3. *All* people have *personal skills and resources*, even if it is difficult for them to identify these, or they are quite minor.
4. Many people are *unaware* of their strengths and resources, and will benefit from having someone help them *think about how they can draw on their support networks and experiences of overcoming problems* in the past.

**Activity 1: Family & Support Network Mapping**

This is an interactive activity that helps the caregiver identify their strengths and support networks. Using the blank page below, ask the caregiver to draw themselves in the middle, and then to add the child(ren) that s/he is caring for. Then ask the caregiver to start adding in people that they feel close to. “Close” can mean people who live in the household or nearby, or close in terms of relationship (people they feel they can trust and rely on).

They should draw these other people on the paper to represent the level of closeness they feel. So a sister or a good friend might be near the centre of the paper, while a teacher or priest from the church might be further toward the edges of the paper. You can ask them questions to prompt whether they have forgotten anyone, e.g. “anyone else?” or “what about members of your church?” or “do you have contact with your own parents?” The caregiver should keep adding people to the page until they have “mapped” their social environment. [Let them draw their own picture – encourage them not to be shy, even symbols or dots are enough!)

Then you should ask, “which of these people help you out in some way?” or “what kinds of favours has this person done for you?” Make a note for each person on the map mentioned by the caregiver, and circle those whom are the most helpful or provide the most support. Ask for specific examples. Then use the map to show that the caregiver and child are not alone or isolated – they have a network of people surrounding them, and will be able to draw on different people for different kinds of support. Maybe some people have not provided any help *yet* but could be relied on in future. Leave the map for the caregiver to keep, and maybe discuss it in future, when problems come up and you want to help the caregiver think about which people may be able to help or support them (and which they can trust enough to disclose to).

**Activity 2: Identifying Strengths, Skills & Resources**

This activity focuses on people’s strengths and positive experiences. Throughout home visits, if you emphasise how caregivers are able to solve problems, you will build their motivation and confidence. Talking about people’s **strengths** is empowering, and helps avoid worrying about barriers and challenges. Using the table below, ask the caregiver to list times from the past when s/he faced a challenge or problem, no matter how small. Note down what s/he did to deal with the problem. In the final column, help him/her identify what **skills** or **resources** were used in that situation to cope, and point out that using these skills and resources will be useful for confronting issues related to HIV treatment and adherence.

Ask the caregiver to keep the table. You can take it out to remind the caregiver that s/he has strengths and skills to cope with problems if s/he shows signs of being discouraged or demoralised during future visits.

## 6.6 FAMILY MAPPING (Resource 1)

**
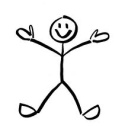
**

**ME**

## 6.7 STENGTHS & SKILLS (Resource 2)

| **Problem faced** | **What I did to overcome it** | **Skills & resources** |
| --- | --- | --- |
|  |  |  |
|  |  |  |
|  |  |  |
|  |  |  |

## 6.8 TIPS FOR POSITIVE LIVING

### 6.8.1 Counselling for Positive Living

As a Lay Health Worker, is important to be familiar with aspects of positive living, as some of the families you visit will be recently diagnosed and may ask you for general advice regarding living with HIV. Some of the children will not be eligible for ART yet, so you many need to explain to the caregiver that this means the child’s immune system is still strong, and it is important to take actions that will keep the child healthy for as long as possible. Once a child starts ART, s/he also needs to follow the principles of healthy *positive living* in order to support treatment.

### 6.8.2 What is positive living?

Positive living is a lifestyle adopted by a person with HIV in order to live life as fully as possible while slowing progression of the disease. It includes making positive choices to care for one’s mental and physical health, having a positive outlook on life, and avoiding risky behaviours. Positive Living can be adopted by the child but also by the caregiver and other family.

You should discuss and encourage the following points:

**How to Live Positively with HIV**

- **Be informed** – Listen carefully to advice from the clinic and from the Lay Health Worker and ask questions whenever you do not understand something about the child’s condition or medication requirements
- **Take medications as prescribed** – Follow instructions for any medications given (Cotri, ART or others) and take them on time, in the right amounts
- **Participate in schoolwork and activities normally, as your energy allows** – Remain involved in usual school, work and leisure activities and participate in social events unless you start to feel too tired or ill
- **Avoid stress** – Everyone suffers stress and anxiety in their lives, but it helps to talk to a friend or a relative of even the LHW! Make time for socialising, relaxing, and try to stay focused on all the positive things in your life.
- **Maintain good nutrition** – Eat a balanced diet and try to eat a little even if you are not feeling well. Check whether any medication you take should be swallowed with food or on an empty stomach. Ask for advice if you are not sure how to help a child eat healthily!
- **Prevent infections** – Maintain good hygiene at home, ensuring everyone washes their hands before food preparation or eating, and after going to the toilet. Wash fruit and vegetables and drink boiled water. Make sure food is fully cooked before eating.
- **Get regular exercise** – Living with HIV does not mean avoiding physical activity! Dancing, running, playing, skipping, participating in sports at school are all very good for the body and the mind! Exercise can also increase appetite and energy levels. But resting enough is also important.
- **Seek regular medical care** – Make sure all clinic appointments are attended, and seek additional medical attention or advice if the child’s health seems to get worse or changes.

## 6.9 DEVELOPING A PERSONAL TREATMENT PLAN FOR CHILDREN

- This tool is designed to facilitate your conversation with the *primary caregiver* of the child you are supporting in treatment adherence and retention in care.
- The steps below should help the caregiver *plan for success* by *thinking* *in advance* of any barriers or challenges that they might face in ensuring adherence to the child’s ART. By planning ahead and putting some practical actions or strategies into place the caregiver might be able to avoid feeling overwhelmed and unable to respond when difficulties arise.
- As a Lay Health Worker, your responsibility is to *suggest* ways to integrate ART into their and the child’s daily lives, *identify* possible obstacles, and *discuss* different options for overcoming these obstacles.
- You may wish to refer back to the list of *strengths*, *skills* and *resources* that you identified with the caregiver previously. These are exactly what they may need to use in order to develop a realistic treatment plan and follow it!

**STEPS**

1. **Check** that the caregiver understands the instructions that have been given for the medication. S/he should be aware of how many drugs have been prescribed, and what time of day each one should be taken. The caregiver should also be able to describe any other conditions related to taking the medication, such as whether they must be taken at the same time as each other, and whether they require food and/or drink, or should be taken on an empty stomach.
2. **Remind** the caregiver of the importance of adherence. When ART is not taken regularly, not only will the medications not work properly to keep the child healthy, but there is also the risk that the child will build up resistance to the treatment so that it will not work in future either. Even missing 1 dose every 10 days or so can reduce the adherence below recommended levels!
3. **Reassure** the caregiver that you are there to help come up with a personalised treatment plan that will make it as easy as possible for the child to take his or her treatment on time, every day. This plan needs to be realistic, and so needs to be developed by the caregiver him-or- herself, with advice from you. It will be a *working document* – something that can be discussed at every home visit and changed whenever necessary.
4. **Ask** the caregiver to describe and note down *their daily routine.* Give them a blank sheet of paper and a pen; they can draw pictures or write things down, whichever they are more comfortable with. Do NOT do this for them – it is important that the caregiver is an active participant in the activity. Start by saying, “I’d like to understand you usual daily schedule in detail. For example, what do you do when you first wake up? And then?” etc., until you have gone through the entire day, from getting out of bed to going to sleep.
5. **Probe** for details. Does the caregiver go to wash right away after getting up? After dressing, what does he or she do? Is the breakfast prepared each morning in the same way? In the evenings, if the caregiver says that they spend some time watching TV or listening to the radio, is it the same programme every day (such as the news or a children’s programme)? You are looking for daily activities that occur regularly at the same time each day, which could be “linked” to giving medication to the child.
6. **Fill out the Personal Treatment Plan** provided on the next page. First, ask the caregiver to think about activities that occur each day at around the right time for giving the child their medication. This will help connect taking medication to a specific daily event – there are lots of possible examples, so make sure that you find some that will fit the daily schedule for the caregiver and/or child and they feel confident they will remember that the treatment should be taken at the same time. Possible routine active `21ties include: eating breakfast, brushing teeth, coming home from school and having a snack, doing maths homework, watching a particular programme, washing dishes, playing with a big sister or brother, reading before bed. List the selected “linked activities” in the Columns 1-3 of the plan.
7. Next, **identify additional reminders**. Although the caregiver might feel confident that s/he will remember to give the child his or her medicine at the same time as the linked activity, you can explain that it is very easy to forget, especially at the beginning of treatment when taking the medicine has not yet become a “routine.” It is useful to also rely on other methods of remembering, or even asking other household members to be part of the treatment plan. Some additional reminders could include setting an alarm on the mobile phone, asking an older sibling to check whether the child has taken the medication (the more people who know that the medicine should be taken at the same time as the “linking activity,” the better!), or writing a note and putting it somewhere that it will be seen easily at the time the medicine is to be taken. List additional reminders in Column 4.
8. Be sure to write down linking activities and additional reminders for *all* the times of the day that medication needs to be taken. **Review the treatment plan** with the caregiver and check that they feel it is realistic.
9. Now **start a conversation** about possible obstacles or challenges that could interfere with successful implementation of the plan. What will happen if there are visitors in the home who do not know the child’s status? Does the caregiver ever travel away from home, and if so, who will ensure the child takes medicines on time? Is the daily routine different on weekends or during school holidays? Really **probe** to find out what situations could arise that would make it difficult for the caregiver to ensure they follow the schedule. Write these down in Column 1 of the Possible Barriers section.
10. Finally, discuss what strategies the caregiver will use to overcome these obstacles. This will involve thinking about strengths, skills and resources, particularly social resources such as friends, family members, neighbours, school teachers, etc., who can step in and support the child’s treatment. If the daily schedule is different at weekends, maybe Saturday and Sunday need different linking activities and reminders. If the caregiver plans to travel, s/he should identify other people who can be trusted to give the child medicine on time or can telephone at those time to remind an adult (or an older child) to provide medication, and then call back later to check. If other people are visiting who do not know about the treatment, the caregiver can make an excuse to leave the room and call the child to talk about something away from the visitors, and give the medicine there, etc. These are just suggestions – you and the caregiver should identify approaches that will be realistic within the situations they are likely to face.

- Remember to **review the treatment plan** at each subsequent home visit. How is it working? Has the child been taking all their doses on time? Every day? Check that the linking activities occur at the same time as the medicine is taken. If not, ask the caregiver to choose a new linking activity to make sure medicine is always associated with a daily task or event. Check if the reminders are working or whether new ones need to be developed. Ask if other people can be included in the plan, to help create a supportive household for the child’s treatment. *The Treatment Plan can be modified or re-written at any time so that it is a useful document!*

____________________________________ **‘s Daily Treatment Plan [Enter child’s name] EXAMPLE COPY**

| **Time of Day** | **Medication required**  **(including dose)** | **Linking Activities** | **REMINDERS**  **(including other people**  **who are involved)** |
| --- | --- | --- | --- |
| Example:  Early morning | Cotri and 3TC | - Preparing porridge first thing after waking up - Giving porridge to children | When niece comes to the house to walk with the children to school, she will ask whether medication has been taken (at the same time that she checks they have their homework.) |
|  |  |  |  |
|  |  |  |  |
|  |  |  |  |

| **POSSIBLE OBSTACLES and…** | **STRATEGIES TO OVERCOME THEM** |
| --- | --- |
| Example:  During the holidays, the children are looked after by caregivers younger sister in the countryside, and do not eat porridge together and do not leave the house with the niece. | The caregiver will explain the times and drugs to her sister, whom she trusts. She will ask her sister to identify a “linking activity” for the mornings during the holidays, such as when the child helps prepare food in the kitchen after getting up. The caregiver will also SMS her sister every day to check that the medication was given on time. She will set her phone alarm to remember to send the message. |
|  |  |
|  |  |

____________________________________ **‘s Daily Treatment Plan [Enter child’s name] COPY FOR CLIENT TO KEEP**

| **Time of Day** | **Medication required**  **(including dose)** | **Linking Activities** | **REMINDERS**  **(including other people**  **who are involved)** |
| --- | --- | --- | --- |
|  |  |  |  |
|  |  |  |  |
|  |  |  |  |
|  |  |  |  |

| **POSSIBLE OBSTACLES and…** | **STRATEGIES TO OVERCOME THEM** |
| --- | --- |
|  |  |
|  |  |
|  |  |
